# Feasibility and preliminary efficacy for morning bright light therapy to improve sleep and plasma biomarkers in US Veterans with TBI. A prospective, open-label, single-arm trial

**DOI:** 10.1371/journal.pone.0262955

**Published:** 2022-04-14

**Authors:** Jonathan E. Elliott, Alisha A. McBride, Nadir M. Balba, Stanley V. Thomas, Cassandra L. Pattinson, Benjamin J. Morasco, Andrea Wilkerson, Jessica M. Gill, Miranda M. Lim

**Affiliations:** 1 VA Portland Health Care System, Research Service, Portland, Oregon, United States of America; 2 Department of Neurology, Oregon Health & Science University, Portland, Oregon, United States of America; 3 Department of Behavioral Neuroscience, Oregon Health & Science University, Portland, Oregon, United States of America; 4 Department of Medicine, Creighton University, Omaha, Nebraska, United States of America; 5 University of Queensland, Institute for Social Science Research, Queensland, Australia; 6 National Institutes of Health, National Institute of Nursing Research, Bethesda, Maryland, United States of America; 7 VA Portland Health Care System, Mental Health, Portland, Oregon, United States of America; 8 VA Portland Health Care System, Center to Improve Veteran Involvement in Care, Portland, Oregon, United States of America; 9 VA Portland Health Care System, Mental Illness Research Education and Clinical Center, Portland, Oregon, United States of America; 10 Department of Psychiatry, Oregon Health & Science University, Portland, Oregon, United States of America; 11 Pacific Northwest National Laboratory, Division of Pulmonary and Critical Care Medicine, Department of Medicine, Oregon Health & Science University, Portland, Oregon, United States of America; 12 Oregon Health & Science University, Oregon Institute of Occupational Health Sciences; Portland, Oregon, United States of America; 13 VA Portland Health Care System, Neurology, Portland, Oregon, United States of America; 14 VA Portland Health Care System, National Center for Rehabilitative Auditory Research, Portland, Oregon, United States of America; Prince Sattam Bin Abdulaziz University, College of Applied Medical Sciences, SAUDI ARABIA

## Abstract

Mild traumatic brain injury (TBI) is associated with persistent sleep-wake dysfunction, including insomnia and circadian rhythm disruption, which can exacerbate functional outcomes including mood, pain, and quality of life. Present therapies to treat sleep-wake disturbances in those with TBI (e.g., cognitive behavioral therapy for insomnia) are limited by marginal efficacy, poor patient acceptability, and/or high patient/provider burden. Thus, this study aimed to assess the feasibility and preliminary efficacy of morning bright light therapy, to improve sleep in Veterans with TBI (NCT03578003). Thirty-three Veterans with history of TBI were prospectively enrolled in a single-arm, open-label intervention using a lightbox (~10,000 lux at the eye) for 60-minutes every morning for 4-weeks. Pre- and post-intervention outcomes included questionnaires related to sleep, mood, TBI, post-traumatic stress disorder (PTSD), and pain; wrist actigraphy as a proxy for objective sleep; and blood-based biomarkers related to TBI/sleep. The protocol was rated favorably by ~75% of participants, with adherence to the lightbox and actigraphy being ~87% and 97%, respectively. Post-intervention improvements were observed in self-reported symptoms related to insomnia, mood, and pain; actigraphy-derived measures of sleep; and blood-based biomarkers related to peripheral inflammatory balance. The severity of comorbid PTSD was a significant positive predictor of response to treatment. Morning bright light therapy is a feasible and acceptable intervention that shows preliminary efficacy to treat disrupted sleep in Veterans with TBI. A full-scale randomized, placebo-controlled study with longitudinal follow-up is warranted to assess the efficacy of morning bright light therapy to improve sleep, biomarkers, and other TBI related symptoms.

## Introduction

Traumatic brain injury (TBI) is a public health concern associated with staggering functional and economic burden, especially in the Veteran population, contributing to a plethora of short- and long-term sequelae [1]. Principal among these complications are sleep-wake disturbances [2–4]. TBI-related sleep-wake disturbances have been documented several years post-injury [5], with recent work suggesting persistence of symptoms >20 years post-injury [6–8]. Multiple mechanisms may explain the pathophysiology underlying persistent sleep disturbances after mild TBI, including but not limited to, damage to cortical pathways related to glutamate/GABA balance and/or orexin/hypocretin neurons, with subsequent effects on circadian-regulated systems such as melatonin, and others [9–13].

Sleep is critical for optimal brain function and general health, including cognition, mood, and resilience to chronic pain [14–18]; especially following TBI [19,20]. Thus, sleep problems in TBI almost certainly exacerbate dependent functional outcomes such as cognition, mood, and chronic pain [21–23]. Current interventions to treat sleep-wake disturbances in those with TBI are limited by marginal efficacy, poor patient acceptability, and/or high patient/provider burden (e.g., medications, acupuncture, cognitive behavioral therapy for insomnia) [24–26]. In particular, Veterans are a unique population with not only high rates of TBI, but also high rates of mental health comorbidities and are also at higher risk for cognitive impairment, both of which may limit the number of treatment options available. Thus, alternative treatment approaches are needed in this population, particularly treatment options that are feasible, have high acceptability, are low burden, and have minimal side effects.

Light therapy has garnered more attention recently due to the possibility of circadian rhythm disruption in TBI as well as effects on sleep, mood and daytime alertness [27–37]. Recent work from Killgore *et al*. in 2020 compared the effects of blue spectrum light with a placebo control amber light on sleep in individuals with TBI, and found improvement in subjective daytime sleepiness, although objective measures of sleep via actigraphy were unchanged [38,39]. Two other recent studies examined light therapy on daytime fatigue in individuals with TBI (including severe) [40,41]. Additionally, light therapy was also recently shown to improve post-traumatic stress disorder (PTSD) symptom severity in Veterans with PTSD, albeit without a change in self-reported or actigraphic metrics of sleep quality [42]. However, to our knowledge, no studies on light therapy have examined effects on sleep in Veterans with TBI. Light therapy is delivered at home and requires little cognitive burden and therefore may be more widely applicable and acceptable to those with cognitive impairment, as is commonly reported in Veterans with TBI [43]. Finally, light therapy has few adverse side-effects, other than select pre-existing conditions (e.g., bipolar disorder and macular degeneration), and thus may be preferable to pharmacologic approaches [44].

We sought to examine the feasibility and preliminary efficacy of light therapy, using a lightbox to deliver white light (10,000 lux at the eye) in the morning, over 4-weeks. The chosen brand is commercially available, has been in use for decades, and was validated with regard to illuminance measures for this study. The chosen dose, timing, and duration is established for seasonal affective disorder and has been shown to affect circadian entrainment [30,44–46], but direct effects on sleep in the Veteran TBI population have not yet been examined. Thus, in addition to feasibility/limited efficacy outcomes, the current study sought to provide preliminary evidence for light therapy to improve sleep in Veterans with a history of TBI. We hypothesized that morning bright light therapy would be feasible, acceptable, and show improvement in both subjective and objective sleep in Veterans in the chronic phase of recovery from mild TBI.

## Materials and methods

**Fig 1:**
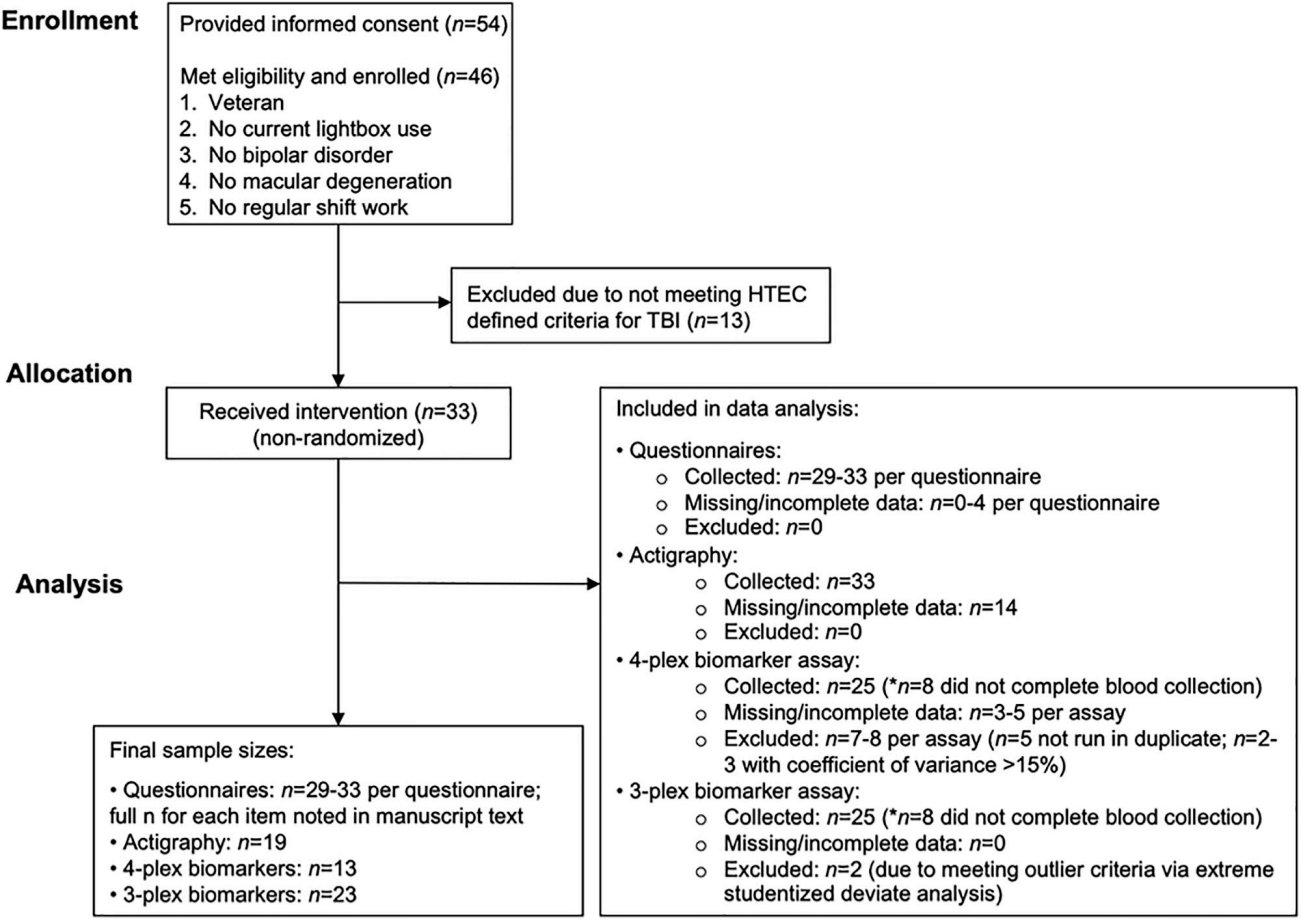
CONSORT diagram. The CONSORT diagram outlining the recruitment, flow, data processing and final *n*’s for select key outcome measures.

The VA Portland Health Care System approved this study, and each subject gave written and verbal informed consent prior to participation (IRB#4085). In this prospective study, Veterans (*n* = 54) were identified from the VA Portland Health Care System Sleep Clinic between 08/2017 and 08/2018. Subjects were excluded if they were not Veterans, currently diagnosed with bipolar disorder, dementia, depression, or macular degeneration, and were either currently using a lightbox or a shift-worker (*n* = 8). No subjects reported using melatonin, and the use of other sleep medications were not excluded for. Eligible subjects (*n* = 46) were consented and further evaluated for TBI by a licensed physician using the Head Trauma Events Characteristics (HTEC; recommended by the Department of Defense and the Department of Veteran Affairs [47]), consisting of a ~20-minute diagnostic interview. In total, *n* = 13 were excluded for not meeting HTEC-defined criteria for sustaining mild TBI. The remaining *n* = 33 subjects, unless otherwise noted, were included in subsequent analyses (Fig 1). This single-arm open-label trial was registered on clinicaltrials.gov as NCT03578003 and presented in accordance with the CONSORT extension for pilot and feasibility trials guidelines [48–50].

### Overview

All subjects followed an identical protocol in this single-arm, open-label study. The baseline period was 7-days, where subjects were instructed to not alter their normal daily routine, including their sleep-wake schedule. Following this, subjects were instructed to receive 60-minutes of bright light therapy (LightPad Mini, Aurora Light Solutions Inc., Reno, NV, USA) every day for 28 consecutive days. Self-report questionnaires were administered pre- and post-intervention, and wrist actigraphy (Actiwatch-2, Philips Respironics, Bend, OR, USA) was collected continuously (35-days). Daily study diaries noting bedtime, wake time, daytime naps, prolonged nocturnal awakenings (>15–30 minutes), and the timing/duration of light therapy were recorded.

### Light therapy

Study personnel provided verbal and visual explanation of how to set-up and turn on/off the lightbox, including correctly positioning the distance (no further than 25-inches), angle (~45°) and pitch (variable) relative to the subject’s face, increasing the intensity to its maximum level, and personalizing use of the device based on subjects’ home configurations. Subjects were permitted to engage in specific activities that did not require them to move away from the lightbox or otherwise avert their eyes or substantially change the direction of their gaze. These activities principally included using a computer or reading. The Aurora LightPad Mini, per the manufacturer and confirmed by our own independent photometer assessments (Dr.meter LX1010B, London, England), produced up to 10,000 lux at the eye, at a distance of 25-inches. Thus, subjects were instructed to keep the lightbox no less than 25-inches from their face. A printed infographic illustrating the above points was attached to each lightbox for constant reinforcement.

### Light validation and reporting

In line with guidance on reporting light exposure in human chronobiology and sleep research studies,[51] we characterized the spectral power distributions (SPD) and illuminance reaching the eye of the participants when using the Aurora LightPad Mini (Table 1). SPDs were recorded using a calibrated spectrophotometer (Konica Minolta, CL-500A Spectrophotometer, NJ, USA; calibrated on 09/09/2019), after allowing the lightbox 10-minutes for warm-up. Spectral radiance measurements were at a distance of 6-inches from the light source, an appropriate distance given the size of the LED light source (5.25” H x 7.5” L; 10x18 LED array), to minimize the influence of reflected light on the SPD measurement. Spectral irradiance estimates were made in an idealized set-up representing how subjects were instructed to position the lightbox, e.g., keeping it at an ~45° angle relative to their field of vision, and angled toward their eyes (generally upward). Ambient light was minimized during these measurements to characterize the output/stimulus provided by the lightbox. Illuminance was recorded using a calibrated illuminance meter (Konica Minolta, T-10A Illuminance Meter, NJ, USA; calibrated 12/17/2018) at a distance of 25-inches reflecting the maximum distance communicated to subjects in dark ambient conditions. The lightbox was positioned at the edge of a table to minimize any reflections off other surfaces before the light reached the detecting source. This series of SPD and illuminance measurements was made on three different lightboxes, with data presented as a group average (S1 Table).

**Table 1:** Aurora LightPad Mini characteristics.

	Properties
Photopic: ƛ_max_, (nm) and illuminance (lux)	555 and 8,763
Irradiance (W/m^2^)	28.66
Log photon irradiance/s/m^2^	19.89
S-cone: ƛ_max_ (nm) and ɑ-opic (W/m^2^)	419 and 7.01
M-cone: ƛ_max_ (nm) and ɑ-opic (W/m^2^)	531 and 12.47
L-cone: ƛ_max_ (nm) and ɑ-opic (W/m^2^)	558 and 14.16
Rhodopic: ƛ_max_ (nm) and ɑ-opic (W/m^2^)	496 and 11.73
Melanopic: ƛ_max_ (nm) and ɑ-opic (W/m^2^)	480 and 10.50
Peak Wavelength, nm	455
Chromaticity, x/y	0.314, 0.329

### Actigraphy

Wrist actigraphy was continuously collected in 2-min epochs for all 35-days. Subjects wore the actiwatch on their non-dominant wrist. Ensuring the photometer was exposed to the light source during treatment (e.g., not under long sleeves, not underneath a table, in a pocket, or otherwise obstructed) was a particular priority. While light detection at the wrist is not the same as light detection at the retina, it provides a crude estimate of adherence, as well as the timing and dose of subject’s light exposure. Actigraphy data were analyzed using the Actiware version 6.0.9 proprietary algorithm (Philips Respironics, Bend, OR, USA) with the activity threshold set to “medium”. Actigraphy based outcomes have been validated against in-lab polysomnography [52]. Each study day, from 12:00 PM to 11:58 AM was analyzed individually for bedtime, sleep onset, wake time, mid-sleep time, total sleep time (TST), time in bed (TIB), sleep onset latency (SOL), sleep efficiency (SE), wake after sleep onset (WASO), total activity, average activity/epoch, and number of nocturnal awakenings. Actigraphy metrics came from data averaged across 3–5 consecutive final days before the pre- and post-intervention assessments. To minimize heterogeneity within subjects, sleep diaries were examined for days where subjects reported not working, working aberrant schedules, or when ill or traveling; these periods were excluded from analyses. On average, the aforementioned sleep diary assessment resulted in excluding 1–2 days over the 35-day study period per subject.

### Questionnaires

#### Sleep

Sleep quality outcomes consisted of the Insomnia Severity Index (ISI) and the Sleep Hygiene Index (SHI). Together these assessments probe difficulty initiating and maintaining sleep, and common behavioral habits contributing to sleep disruption. The ISI is a 7-item measure, each item a 5-point Likert scale [53,54]. The SHI is a 13-item measure, each item a 5-point Likert [55].

#### TBI and PTSD

TBI-relevant outcomes were the Neurobehavioral Symptom Inventory (NSI) and the PTSD checklist for DSM-5 (PCL-5). The NSI assesses TBI relevant symptom severity over the past 2-weeks, and is composed of 22-items, each a 5-point Likert scale [56–58]. The PCL-5 is used for screening/provisional diagnosis of PTSD, and assessing symptom severity over the 30-days using 20-items, each a 5-point Likert [59]. The total score can be subdivided into four subscales or “clusters”: Cluster B (intrusion), cluster C (avoidance), cluster D (mood/cognition), and cluster E (arousal). PTSD was determined by a total score ≥33 and meeting “cluster criteria”, as before [6–8], requiring subjects to rate one B item, one C item, two D items, and two E items as 2 (“moderately”) or higher [59].

#### Mood

Metrics related to mood were derived from the Patient Health Questionnaire (PHQ-9) and NIH PROMIS Emotional-Distress and Anxiety (EDA) short-form 4a. The PHQ-9 assesses mood and depression severity over the previous 2-weeks. It is 9-items, each a 4-point Likert scale. The EDA comes from the NIH PROMIS catalog and assesses anxiety severity over the past 7-days. It is composed of 4-items, each a 5-point Likert scale.

#### Pain

Metrics of pain come from the NIH PROMIS Pain Interference short-form 4a and the Pain Intensity short-form 3a, assessing perceived pain over the past 7-days. Pain Interference is composed of 4-items, each a 5-point Likert scale. Pain intensity is composed of 3-items, each a 5-point Likert scale.

#### Quality of life

The World Health Organization Disability Assessment Schedule (WHO-DAS 2.0) assesses general health and disability in major life domains following the conceptual framework for the International Classification of Functioning, Disability, and Health over the past 30-days. It is composed of 12-items, each a 5-point Likert scale.

### Blood-based biomarkers

Whole blood was collected pre- and post-intervention (i.e., generally day 1 and 35 of the protocol, corresponding to the beginning of baseline and end of intervention), immediately inverted 10x and stored at 4°C until processing for plasma and serum (~1–3 hours). Aliquots were stored at -80°C until a sufficient number was obtained for batch assays. Plasma samples were sent to the NIH NINR Biomarker Core Laboratory (PI: J.M.G.) for 4-plex and 3-plex immunoassays (exploratory outcomes) using an ultrasensitive single-molecule enzyme-linked immunosorbent assay via Simoa^™^ technology (Quanterix, Lexington, MA, USA) [60–62]. The 4-plex measured concentrations of glial fibrillary acidic protein (GFAP), neurofilament light chain (NfL), tau, and ubiquitin C-terminal hydrolase-L1 (UCHL1) [63]. The 3-plex measured concentrations of interleukin-6 (IL-6), interleukin-10 (IL-10), and tumor necrosis factor-alpha (TNF-ɑ).

Of the *n* = 33 subjects, pre- and post-blood samples were obtained in *n* = 25 subjects. For the 4-plex assay, a final *n* = 13 was used (12-subjects excluded due to either unreadable results (*n* = 3 to 5 per assay), data not being obtained in duplicate (*n* = 7 to 8 per assay), or having a coefficient of variance >15% (*n* = 2 to 3 per assay) (Fig 1). UCHL1 was detectable for only 4 subjects (2 of which were not obtained in duplicate), as such these UCLH1 data were excluded. No outliers were detected using the extreme studentized deviate analysis. For the 3-plex assay, a final sample size of *n* = 23 was used (2 subjects excluded due to meeting outlier criteria). The average coefficients of variance for the 4-plex and 3-plex assays were, 4.5±3.0% and 3.2±2.5%, respectively.

### Statistical analysis

Analyses were performed using GraphPad Prism v8.4.2. Alpha of 0.05, defined *a priori*, was used for all tests unless otherwise noted. Mean differences between pre- and post-intervention outcomes were assessed via paired, two-tailed t-tests. Pearson *r* correlation coefficient, and corresponding confidence intervals, were analyzed comparing the percent improvement in ISI score (primary outcome) to ancillary outcome measures (*Questionnaires*: SHI, PCL-5, NSI, PHQ-9, EDA, Pain intensity, Pain interference, WHO-DAS 2.0. 2. *Actigraphy*: TST, TIB, SE, WASO, SOL. *Blood-based biomarkers*: GFAP, NfL, tau, IL-6, IL-10, TNF- ɑ). Subsequently, exploratory multiple linear regression analyses were performed to parse out the most impactful contributing variables to subjects percent improvement in ISI score. Models included 1) all questionnaires with a significant post-intervention change, 2) significant actigraphy derived metrics, and 3) significant blood-based biomarker changes (including additional combinations of models encompassing all variables, and models with hierarchal inclusion). As a final measure of association, the odds ratio was used. These models were strictly exploratory and should be interpreted as such.

## Results

Demographic and TBI-related data are presented in Table 2. This population was predominantly middle-aged, male, white, with at least some college education, and on average exercised >90-minutes/week. All subjects were in the chronic phase of TBI recovery (range: 3–55 years post-injury), with 78% reporting 1–3 TBIs over their lifetime. Forty-two percent of subjects incurred their injury through active military combat. Causes of TBI ranged from blast exposure, blunt force, falling, and motor vehicle or sports-related accidents. Self-reported location of injury was unknown or via blast exposure in 8 subjects, and otherwise determined to be in the frontal (*n* = 7), right temporal (*n* = 3), right parietal (*n* = 2), left parietal (*n* = 3) and occipital (*n* = 10) region. A total of 12 subjects reported loss of consciousness, and 11 subjects reported some degree of post-traumatic amnesia, however, all injuries were determined to be of mild severity (i.e., mTBI). PTSD was present in 18 (54%) subjects.

**Table 2:** Demographic and TBI related variables.

*n*	33
Age, years	53 ± 18
Sex, male	30 (91%)
Race, white	25 (76%)
Education, ≥some college	28 (85%)
Exercise, >90 min/week	23 (70%)
TBI recency, years	26 ± 17
TBI recency range, years	3–55
Number of TBIs	
1	12 (36%)
2–3	14 (42%)
4–5	4 (12%)
>5	3 (10%)
Type	
Blast	4 (12%)
Blunt force	6 (18%)
Fall	12 (36%)
Sports/MVC	9 (27%)
Unknown	2 (6%)
Loss of consciousness	
None	18 (55%)
<30 seconds	6 (18%)
1–5 minutes	5 (15%)
>5 minutes	1 (3%)
Post-traumatic amnesia, yes	11 (33%)
Sleep medication, yes	17 (51%)
Pain medication, yes	19 (56%)
PTSD, yes	18 (54%)

Data are mean ± SD, or *n* (%). TBI, traumatic brain injury; PTSD, post-traumatic stress disorder. Sleep medication includes any of the following: Sedative-hypnotics, benzodiazepines, gamma-hydroxybutyric acid, doxylamine, trazadone, quetiapine, diphenhydramine, mirtazapine, and over the counter herbs. Pain medication includes any of the following: Oxycontin, hydrocodone, morphine, oxycodone, hydrocodone bitartrate, fentanyl patch, methadone, codeine, naltrexone/suboxone, and lidocaine patch.

### Acceptability, feasibility, and adherence

Of the 33 subjects, 22 (67%) reported liking the lightbox, 8 (24%) reported not liking the lightbox, and 3 (9%) neither liked nor disliked the lightbox. Reasons for not liking the lightbox were predominantly due to the light being too bright and/or the light disturbing other people in the subject’s household. Similarly, 22 (67%) of subjects reported liking wearing the actiwatch, 5 (15%) reported not liking wearing the actiwatch and 6 (18%) neither liked nor disliked wearing the actiwatch. Reasons for not liking the actiwatch were primarily because of discomfort/annoyance.

With respect to treatment feasibility and adherence, 30 (91%) subjects reported using the lightbox 5–7 days/week. Two subjects reported using the lightbox 3–4 days/week, and 1 subject reported using the lightbox 1–2 days/week. Average percent adherence based off of subjects reported daily usage (time of day/duration) logged in their sleep diary was 83% (range: 43%-100%; 29 subjects were >74%). In the majority of cases, photometer data aligned with self-reported use of the lightbox. Discrepancies are assumed to be due to user error (e.g., sleeves covering wrist or watch not facing the lightbox). For the actiwatch, 32 (97%) of subjects reported wearing it 7 days/week and only *n* = 1 subject reported wearing it 5–6 days/week. In general, this closely aligned with subjects’ objective data.

### Sleep

**Fig 2:**
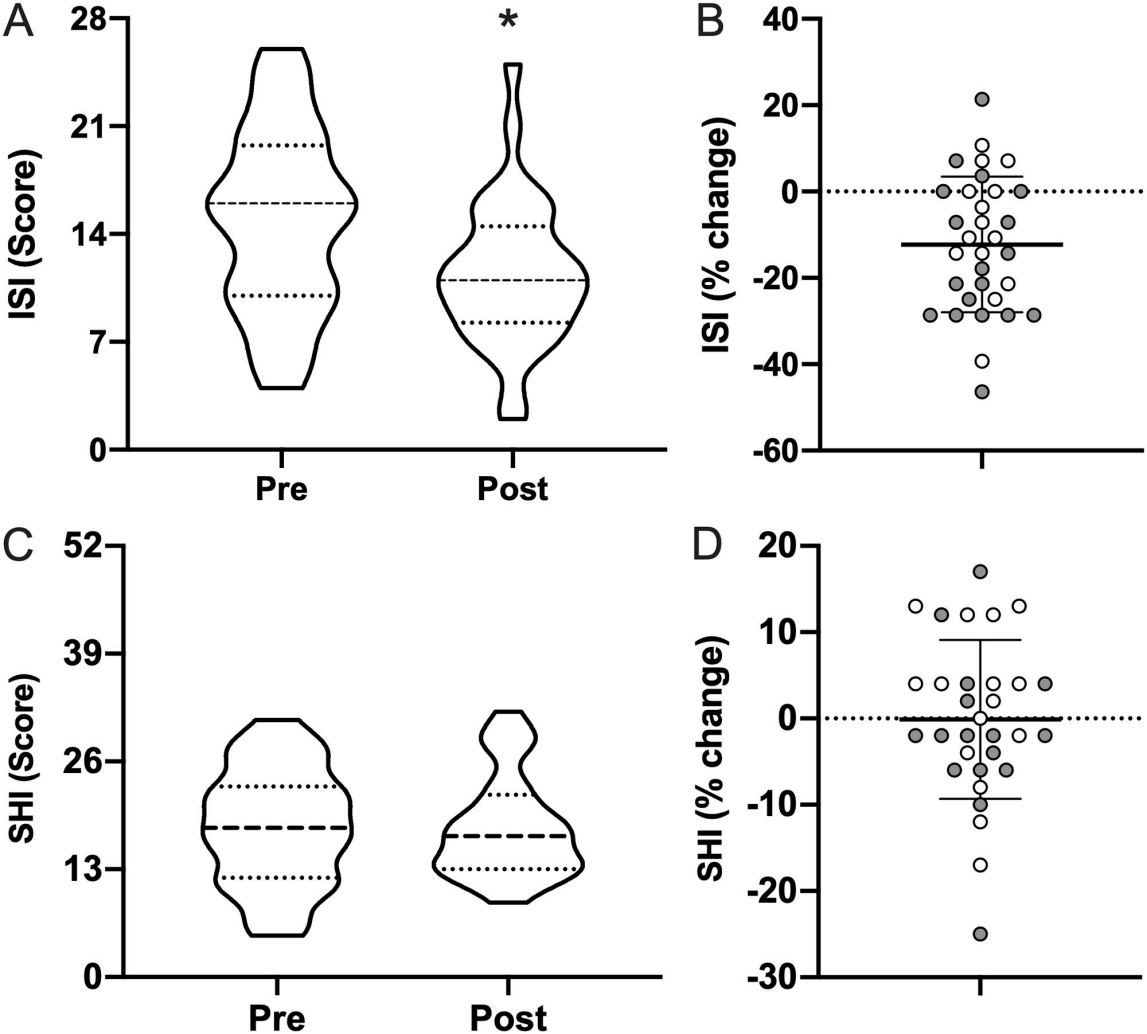
Sleep related questionnaire data. Pre- and post-morning bright light therapy questionnaire scores for the (**A**) Sleep Hygiene Index (SHI; range = 0–52; higher = worse sleep hygiene) and (**C**) Insomnia Severity Index (ISI; range = 0–28; higher = worse insomnia) displayed as truncated violin plots with moderate smoothing (dashed line = median score; dotted lines = 25% and 75% quartiles). Percent change in each Individual for (**B**) SHI and (**D**) ISI, normalized to the maximum score of each questionnaire, plotted with the mean and standard deviation overlaid. Shaded symbols are subjects with co-morbid post-traumatic stress disorder (PTSD), open symbols are subjects without co-morbid PTSD. * = *p* <0.05 vs pre; paired two-tail t-test.

ISI scores improved following light therapy (Fig 2) without a concomitant change in SHI. Subjects ISI score decreased by 23% (14.8±5.9 to 11.4±4.7; *p*<0.0001), clinically corresponding to a change of “moderate” to “mild” insomnia. Pre and post-SHI scores were unchanged (18.1±7.1 to 18.0±6.2; *p* = 0.97).

Objective actigraphic outcomes (Table 3) were consistent with these self-report metrics, with an average improvement in TST of 47-minutes (5:33±1:07 to 6:22±1:13; *p* = 0.004), corresponding to an average increase in TIB of 59-minutes (6:40±1:04 to 7:39±1:13; *p* = 0.001). Normalizing these metrics to individual durations show a 21.2%±27.5% increase in TST, and a 16.3%±19.6% increase in TIB. Other metrics, including SE, WASO, SOL, activity, and the number of nocturnal awakenings were unchanged. The increase in TST and TIB came from subjects shifting their bedtime earlier, by an average of 77-minutes (00:04±1:36 to 22:47±4:24; *p* = 0.001). Waketimes averaged 30-minutes earlier, though this change was not statistically significant (*p* = 0.061), but were found to be more regular (i.e., less variable; p = 0.022). Mid-sleep times were also shifted earlier by an average of 37-minutes (3:13±1:15 to 2:36±1:31; *p* = 0.003).

**Table 3:** Actigraphy metrics pre- and post-morning bright light therapy.

	Pre	Post	*p* value
Bedtime, hh:mm	00:04 ± 1:36	22:47 ± 4:24	**0.001**
Waketime, hh:mm	6:52 ± 1:22	6:22 ± 1:37	0.061
Mid-sleep time, hh:mm	3:13 ± 1:15	2:36 ± 1:31	**0.003**
Time in bed, hh:mm	6:40 ± 1:04	7:39 ± 1:13	**0.001**
TST, hh:mm	5:33 ± 1:07	6:22 ± 1:15	**0.004**
SOL, min	5.4 ± 5.4	6.7 ± 8.2	0.597
SE, min	82.8 ± 8.4	83.3 ± 7.7	0.713
WASO, min	49.2 ± 23.6	59.5 ± 31.7	0.061
Total Activity, units	204091 ± 72912	210932 ± 47386	0.278
Avg AC/epoch	459.0 ± 177.0	531.5 ± 107.0	0.634
Awakenings, #	12.3 ± 5.0	13.1 ± 3.6	0.452
Light exposure, lux-min	250 ± 204.7	38910.3 ± 36180.5	**0.005**

Data are mean ± SD. TST, total sleep time; SOL, sleep onset latency; SE, sleep efficiency; WASO, wake after sleep onset; AC, activity counts. Data were analyzed via paired students t-test. * = *p* <0.05 versus Pre.

### TBI-related symptoms

**Fig 3:**
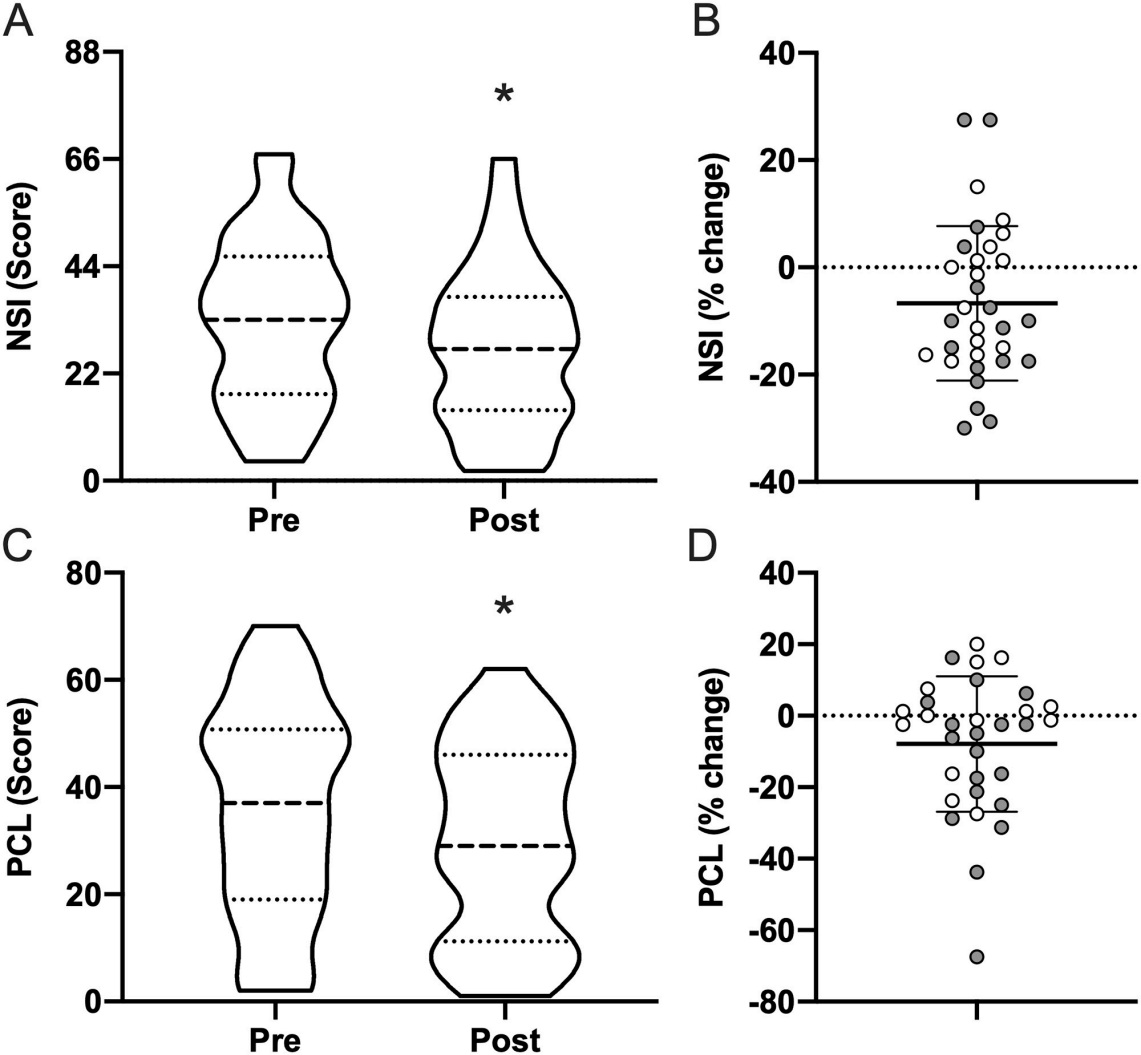
TBI-related symptom severity. Pre- and post-morning bright light therapy questionnaire scores for the (**A**) Neurobehavioral Symptom Inventory (NSI; range = 0–88; higher = worse brain injury related symptom severity) and (**C**) Post-traumatic stress disorder (PTSD) checklist-5 (PCL-5; range = 0–80; higher = worse PTSD symptom severity) displayed as truncated violin plots with moderate smoothing (dashed line = median score; dotted lines = 25% and 75% quartiles). Percent change in each Individual for (**B**) NSI and (**D**) PCL-5 normalized to the maximum score of each questionnaire, plotted with the mean and standard deviation overlaid. Shaded symbols are subjects with co-morbid post-traumatic stress disorder (PTSD), open symbols are subjects without co-morbid PTSD. * = *p* <0.05 vs pre; paired two-tail t-test.

Subjects reported improvements in both the NSI and PCL-5 (Fig 3). NSI scores were reduced by 16% (32.5±17.0 to 27.2±16.4; *p* = 0.013), and PCL-5 scores were reduced by 18% (35.8±19.9 to 29.5±18.2; *p* = 0.025). The percent improvement in ISI scores was positively correlated with subjects’ percent improvement in both the NSI (*r* = 0.39; CI = 0.05–0.66; *p* = 0.028), and PCL-5 (*r* = 0.69; CI = 0.44–0.84; *p*<0.0001). Additionally, percent improvement in ISI was also negatively correlated with subjects’ baseline PCL-5 score, i.e., worse PTSD symptom severity predicted greater improvement in ISI scores (*r* = -0.41; CI = -0.67–0.07; *p* = 0.021).

### Mood symptoms

**Fig 4:**
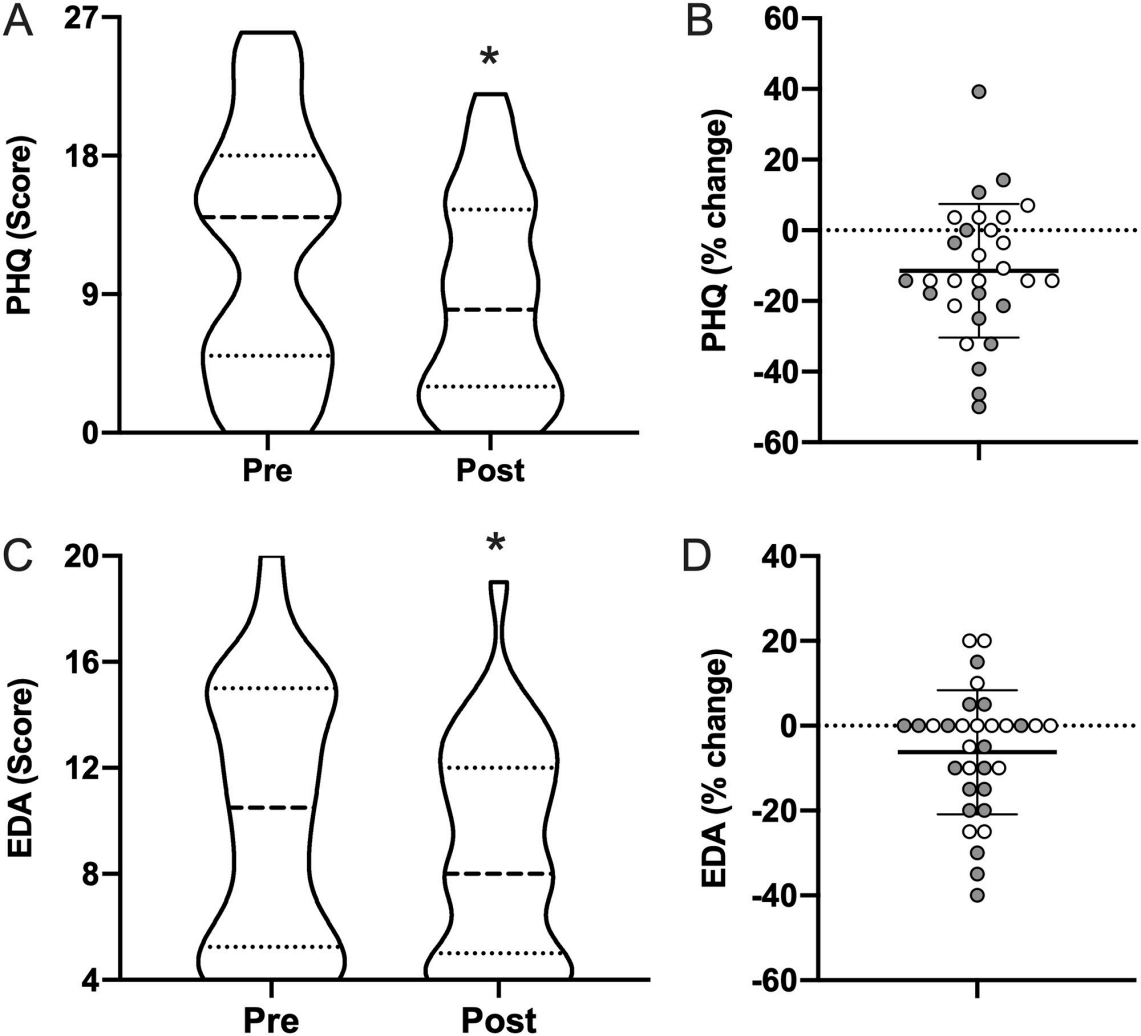
Mood and anxiety. Pre- and post-morning bright light therapy questionnaire scores for the (**A**) Patient Health Questionnaire-9 (PHQ-9; range = 0–27; higher = worse depressive symptom severity) and (**C**) NIH PROMIS Emotional-Distress and Anxiety (EDA; range 4–20; higher = worse symptom severity) displayed as truncated violin plots with moderate smoothing (dashed line = median score; dotted lines = 25% and 75% quartiles). Percent change in each Individual for (**B**) PHQ-9 and (**D**) EDA normalized to the maximum score of each questionnaire, plotted with the mean and standard deviation overlaid. Shaded symbols are subjects with co-morbid post-traumatic stress disorder (PTSD), open symbols are subjects without co-morbid PTSD. * = *p* <0.05 vs pre; paired two-tail t-test.

Both the PHQ-9 and EDA scale showed significant improvements (Fig 4). PHQ-9 scores decreased by 27% (11.9±8.0 to 8.6±6.6; *p* = 0.003), and EDA scores decreased by 12.7% (10.2±4.9 to 8.9±4.0; *p* = 0.021). Percent improvement in ISI was positively correlated with percent improvement in PHQ-9 (*r* = 0.55; CI = 0.22–0.76; *p* = 0.002), but not with percent improvement in EDA.

### Pain symptoms

**Fig 5:**
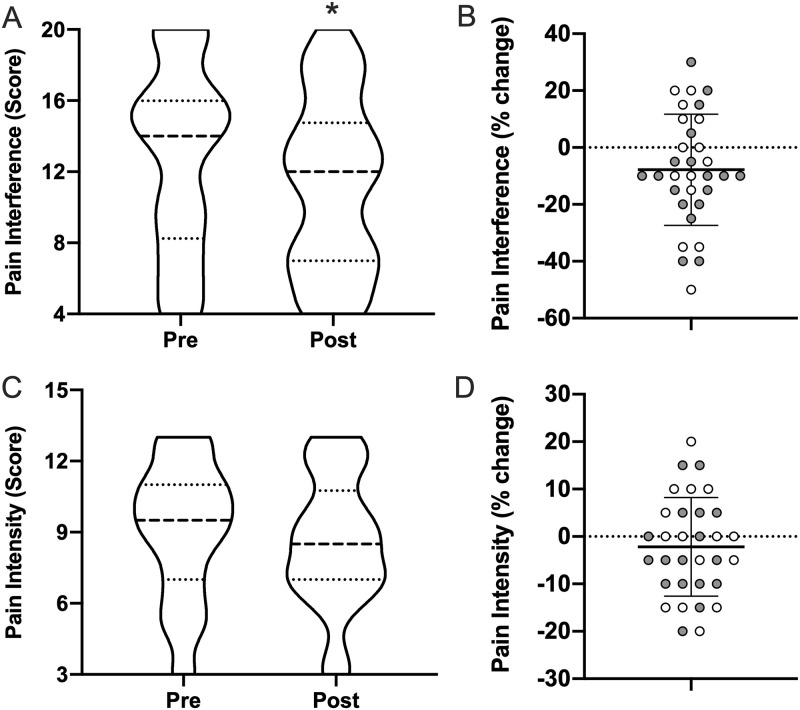
Pain interference and intensity. Pre- and post-morning bright light therapy questionnaire scores for the (**A**) NIH PROMIS Pain Interference (range = 4–20; higher = worse pain interference) and (**C**) NIH PROMIS Pain Intensity (range = 3–15; higher = pain intensity) displayed as truncated violin plots with moderate smoothing (dashed line = median score; dotted lines = 25% and 75% quartiles). Percent change in each Individual for (**B**) pain interference and (**D**) pain intensity, normalized to the maximum score of each questionnaire, plotted with the mean and standard deviation overlaid. Shaded symbols are subjects with co-morbid post-traumatic stress disorder (PTSD), open symbols are subjects without co-morbid PTSD. * = *p* <0.05 vs pre; paired two-tail t-test.

No changes were reported in pain intensity (9.0±2.9 to 8.6±2.8; *p* = 0.24); however, subjects did show a significant reduction in pain interference (12.8±5.2 to 11.2±4.9; *p* = 0.031), corresponding to a decrease of 12.5% (Fig 5). This dissociation between pain intensity and interference (e.g., ability to cope with intensity) is of particular interest as it emphasizes a role for light therapy and improved sleep on functional outcomes, rather than changes to the fundamental biology of nociception. No significant correlations between pain intensity or interference with subject’s percent change in ISI were observed.

### Quality of life

**Fig 6:**
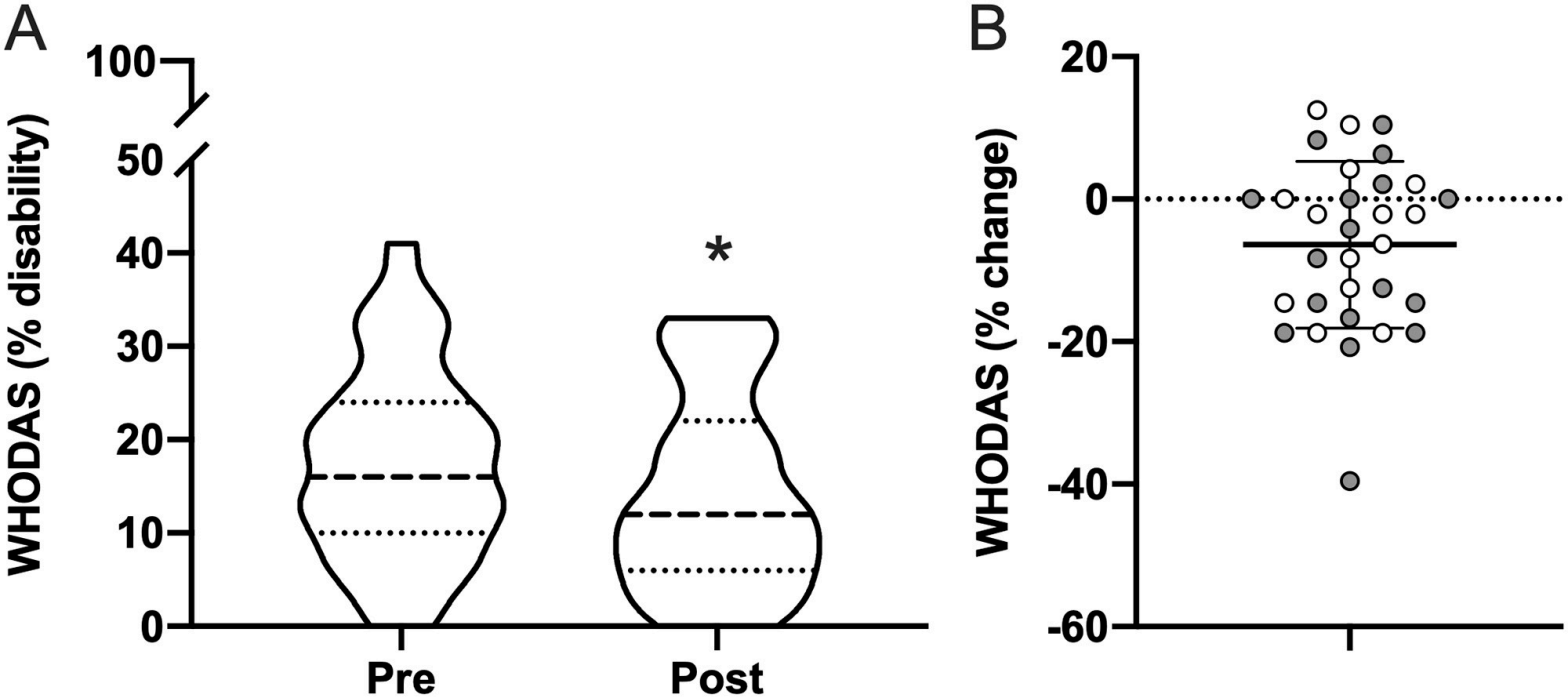
Quality of life. Pre- and post-morning bright light therapy questionnaire scores for the (**A**) World Health Organization Disability Assessment Schedule 2.0 (WHO-DAS 2.0; range = 0–100%; higher = worse quality of life/greater disability) is displayed as a truncated violin plot with moderate smoothing (dashed line = median score; dotted lines = 25% and 75% quartiles). Percent change in each Individual for (**B**) WHO-DAS 2.0 is normalized to the maximum score of this questionnaire, plotted with the mean and standard deviation overlaid. Shaded symbols are subjects with co-morbid post-traumatic stress disorder (PTSD), open symbols are subjects without co-morbid PTSD. * = *p* <0.05 vs pre; paired two-tail t-test.

Subjects reported a significant improvement in the WHO-DAS 2.0 (Fig 6), corresponding to a decrease of 17.7% (36.2%±21.5% to 29.8%±21.8%; *p* = 0.005). No significant correlation between WHO-DAS with subject’s percent change in ISI was found.

### Blood-based biomarkers

**Fig 7:**
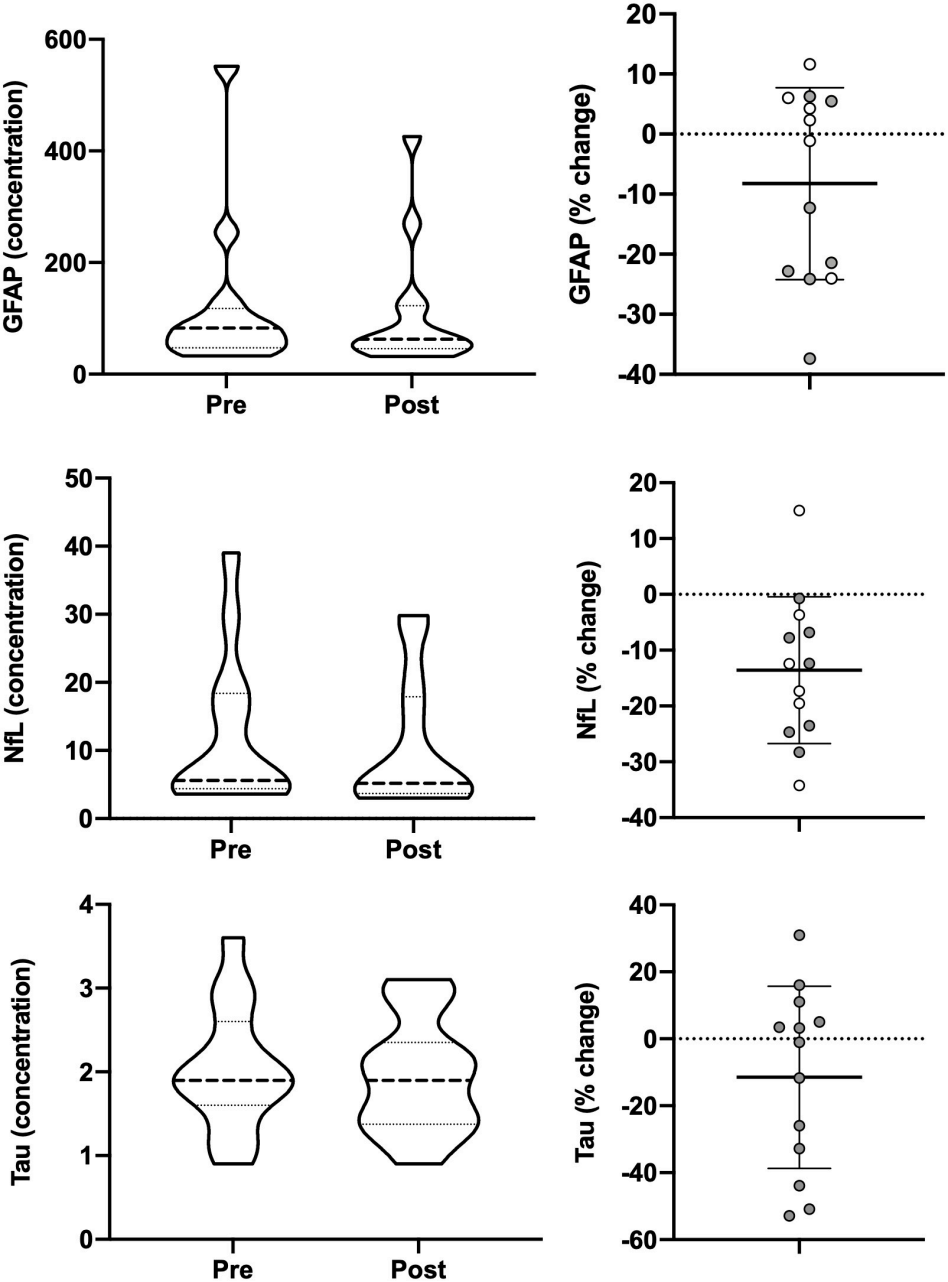
Neuronal injury and neuroinflammation biomarkers. Pre- and post-morning bright light therapy biomarkers for (**A**) Glial Fibrillary Acidic Protein (GFAP), (**C**) Neurofilament Light Chain (NfL), and (**E**) Tau, displayed as truncated violin plots with moderate smoothing (dashed line = median score; dotted lines = 25% and 75% quartiles). Percent change in each Individual for (**B**) GFAP, (**D**) NfL, and (**F**) Tau, plotted with the mean and standard deviation overlaid. Shaded symbols are subjects with co-morbid post-traumatic stress disorder (PTSD), open symbols are subjects without co-morbid PTSD. * = *p* <0.05 vs pre; paired two-tail t-test.

**Fig 8:**
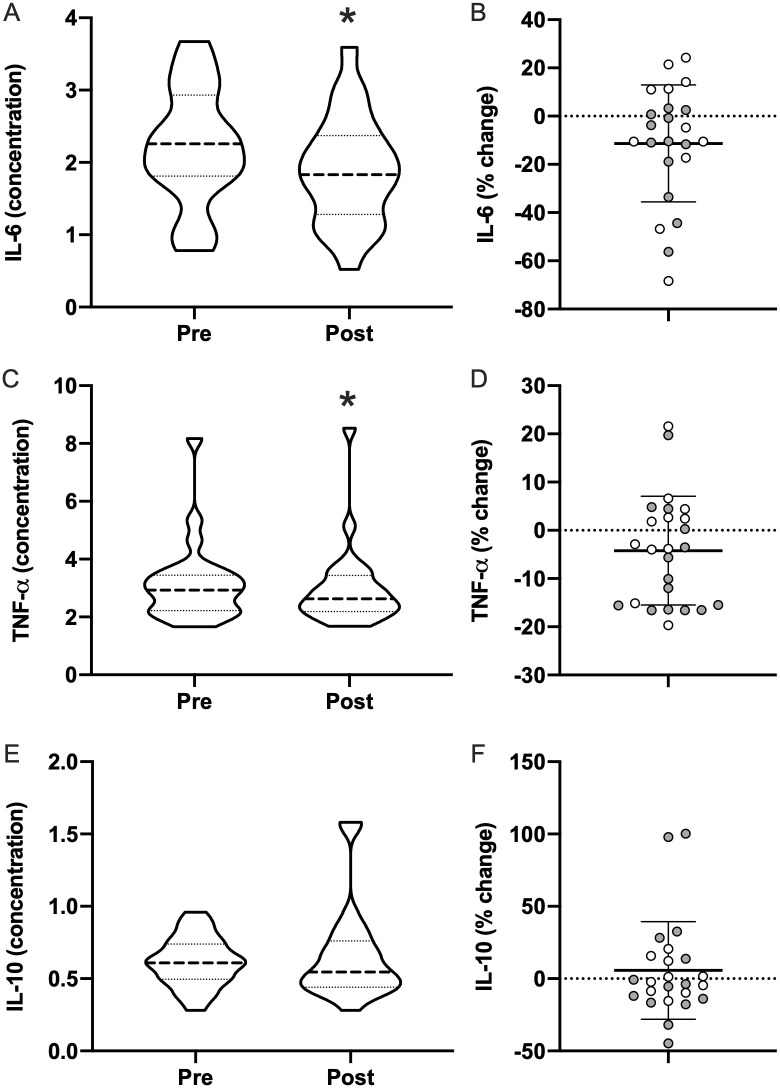
Peripheral cytokines. Pre- and post-morning bright light therapy biomarkers for (**A**) Interleukin-6 (IL-6), (**C**) Tumor Necrosis Factor Alpha (TNF-ɑ), and (**E**) Interleukin-10 (IL-10) displayed as truncated violin plots with moderate smoothing (dashed line = median score; dotted lines = 25% and 75% quartiles). Percent change in each Individual for (**B**) IL-6, (**D**) TNF-ɑ, and (**F**) IL-10, plotted with the mean and standard deviation overlaid. Shaded symbols are subjects with co-morbid post-traumatic stress disorder (PTSD), open symbols are subjects without co-morbid PTSD. * = *p* <0.05 vs pre; paired two-tail t-test.

Changes in plasma biomarkers related to peripheral markers of neuronal injury/neuroinflammation and inflammation were assessed as exploratory outcomes. No differences pre- and post-intervention were observed for GFAP, NfL, and tau (Fig 7); however, trends were detected in NfL showing a decrease of 13.2% (12.1±11.4 to 10.5±9.8; *p* = 0.064), and tau showing a decrease of 17.4% (2.3±0.6 to 1.9±0.7; *p* = 0.103). The pro-inflammatory cytokines IL-6 (2.2±0.8 to 1.8±0.7; *p* = 0.028) and TNF-ɑ (3.1±1.4 to 2.9±1.4; *p* = 0.037) were significantly reduced by 14.5% and 5.7%, respectively, following light therapy (Fig 8). The anti-inflammatory cytokine IL-10 was unchanged (0.6±0.2 to 0.7±0.3; *p* = 0.43). Percent change in ISI was positively correlated with subject’s percent change in IL-6 (*r* = 0.43; CI = 0.02–0.72; *p* = 0.043), but not with percent change in TNF-ɑ or IL-10.

### Contribution of PTSD

Due to the multiple significant correlations identified between percent change in ISI and changes in other self-report measures, multiple linear regression analyses were performed to determine which self-report measures had the largest contribution to these effects. Several models were examined, including a model containing all self-report questionnaires that showed significant changes post-intervention as predictor variables. In all models, percent change in PCL-5 was the sole significant predictor of improvement in ISI after light therapy (β = 0.586; *p* = 0.006).

No significant effect of PTSD was found through multiple linear regression models of either blood-based biomarkers, or actigraphy derived metrics, in predicting subjects’ percent change in ISI score.

To assess the association between change in ISI and PTSD, the odds ratio was calculated separating those without PTSD (*n* = 15) from those with PTSD (*n* = 18). When using a criterion of a ≥4-point decrease in ISI score to define response to light therapy, subjects with comorbid PTSD had 5.76 times the odds (CI: 1.18–28.28; *p* = 0.041) of showing a response to light therapy compared to subjects without PTSD. Although this association should be interpreted knowing that odds ratios are generally overestimated with smaller sample sizes, this association is also supported by the raw data. On average, subjects with co-morbid PTSD had higher pre-intervention ISI scores (Fig 2A; with PTSD, 17.1±5.3 vs without PTSD, 11.9±5.5), as well as a greater percent change post-intervention (Fig 2B; with PTSD, 15.1±16.9% vs without PTSD, 8.7±13.8%), corresponding to post-intervention ISI scores of 12.9±4.5 (TBI with PTSD) vs. 9.5±4.6 (TBI without PTSD). Based on these raw scores, subjects with PTSD shifted from “moderate” insomnia to “mild” insomnia, while those without PTSD remained in the “mild” category.

### Harms

There were no documented important harms or unintended effects throughout the course of this study.

## Discussion

This single-arm, open-label feasibility study using morning bright light therapy in Veterans with TBI shows high patient acceptability, adherence, and preliminary efficacy on both subjective and objective sleep outcomes. Furthermore, downstream mechanisms potentially affected by poor sleep, including mood, pain and quality of life also showed improvement. Blood-based biomarkers showed improvements in IL-6 and TNF-ɑ, peripheral pro-inflammatory cytokines, and trends toward potential improvements in markers associated with neuronal injury and neuroinflammation (NfL and tau). Overall, these results support the rationale for a fully powered randomized controlled trial with longitudinal follow-up to more rigorously evaluate morning bright light therapy as an intervention in this patient population. Having an effective and acceptable intervention to improve sleep disturbances—a persistent and debilitating consequence of TBI—will be critical to adequately address disability and optimize rehabilitation in Veterans in the chronic phase of recovery from TBI.

### Light therapy improved subjective and objective sleep

Our results showed that both subjective (self-report surveys) and objective sleep (wrist based actigraphy) improved with morning bright light therapy. Although improvements in sleep following morning bright light therapy, either subjective or objective, have been reported, this is not a universal finding. A recent systematic review and meta-analysis on the effect of light therapy on sleep problems [64] highlights the importance of the type of sleep problem (e.g., insomnia, circadian rhythm disorders, dementia), the number of treatment days, the duration of treatment, and the age of subjects on determining whether or not a beneficial effect of light therapy is observed. This review incorporates data from 53 studies and via multivariate analyses show an overall hedges g effect size of 0.39 for *all* outcome variables across *all* sleep problems. Hedges g effect sizes shift to 0.41 and 0.47 for *all* outcome variables, when only including studies on circadian rhythm sleep disorders (*n* = 15 studies) and insomnia (*n* = 15 studies), respectively. This review did not discuss the alignment/concordance across subjective and objective sleep outcomes within studies, but it is known that this is also an inconsistent finding in the literature. Alignment between subjective and objective sleep outcomes in the present study lends validity to the potential positive effect of light therapy on sleep in this population. Mean pre-ISI scores were ~15 and decreased to ~11 post-intervention, corresponding to a baseline diagnosis of moderate insomnia (15–21) improving to mild insomnia (8–14).

Using actigraphy as a long-term objective measure of sleep is a strength of the study but brought challenges to implementation and data analysis. For example, employed subjects had variable schedules, which was addressed by manually curating ~5 consecutive days consistent with each subject’s workweek. We found the photometer on the actiwatch an unreliable indicator for lightbox usage. This was likely due to variability in distance and placement of the wrist from the lightbox, and whether subjects’ long-sleeved clothing or other material inadvertently obscured the photometer. Nevertheless, our data indicate the photometer was able to detect higher levels of light in the intervention period compared to the baseline.

### Potential mechanisms of light therapy to improve sleep

Seminal work identified a role for light in melatonin suppression [65,66] and since then, research has focused on the effect of light on shifting circadian rhythms [67]. More recent work has identified a role for light in improving depression, with animal models pointing to mechanisms related to direct neuronal projections from non-vision-forming pathways to limbic regions of the brain [30,31,68,69]. Few studies have found morning bight light therapy effects on sleep directly, although this has not been studied to the extent of the circadian and mood fields [32,70]. Some possibilities include light may have direct effects on brain regions involved in sleep (e.g., direct inhibition of the ventrolateral preoptic area), or maintenance of alertness, through the same melanopsin-dependent, non-image forming pathways as circadian rhythms and mood [71]. Increased alertness throughout the day may increase homeostatic sleep drive in the evening and help to consolidate nighttime sleep [72]. Alternatively, given the known bidirectional relationship between sleep and depression [73], it is also possible that light-induced mood improvement may indirectly improve sleep [31].

This study was not designed to examine the underlying mechanisms of light therapy’s effects on sleep, but could potentially inform mechanistic-based hypotheses. Although this study did not assay melatonin to determine subjects dim light melatonin onset (DLMO; gold standard for assessing a shift in circadian rhythm), subjects mid-sleep times were determined via actigraphy which may be an acceptable surrogate to DLMO in estimating circadian shifts [74,75]. Morning bright light therapy shifted mid-sleep time by an average of 37-minutes, indicating a possible circadian mechanism. However, shifting bedtimes earlier (e.g., advancing circadian phase) may not necessarily increase total sleep time, which was observed. Therefore, while circadian phase advancement may contribute toward improvements in ISI, there are likely other factors as well. Of note, no subjects demonstrated clear evidence of any circadian rhythm sleep disorder on baseline actigraphy.

### Light therapy and blood-based biomarkers

A pipeline for smooth blood collection, processing, storage, and shipment to the NIH Core lab for assays was successfully established; however, we experienced substantial attrition of nearly half the samples with the 4-plex assay (*n* = 13). Furthermore, one of the 4 biomarkers in the 4-plex, UCHL1, was unable to be included in our analyses. However, NfL and tau are both widely accepted markers of neuronal injury [60,76,77]. Further, despite the small sample size, peripheral markers of neuronal injury and neuroinflammation such as NfL and tau still showed trends towards statistical significance in directions consistent with the existing literature.

The 3-plex assay was much more robust, with the majority of the sample retained for analysis (*n* = 23 included). We found reductions in pro-inflammatory cytokines IL-6 and TNF-ɑ, and no change in the anti-inflammatory cytokine IL-10. These results are also consistent with existing literature, as other studies have reported improvements in pro-inflammatory cytokines with improved sleep, including treatment of sleep apnea [60,78].

Of note, peripheral plasma does not reflect centrally circulating biomarkers. Future studies will explore brain-specific biomarkers, such as brain-derived exosomes containing tau and amyloid-β-42; promising markers in the neuropathology of TBI [77].

### Contribution of PTSD to sleep and symptoms

A common theme from this study, and other recent studies of TBI in the military population, is the importance of PTSD [6–8]. Multiple linear regression models in this study showing comorbid PTSD was the sole significant predictor for improved insomnia following light therapy. Additionally, individuals with TBI and comorbid PTSD had >5 times the odds of showing an improvement in ISI score of at least 4-points. Given that the average baseline ISI score in this cohort was ~15 (i.e., moderate insomnia), a decrease in at least 4-points would reduce subjects to mild insomnia. This observation may be relevant for informing clinical decisions as to which subset of TBI patients may benefit the most from lightbox therapy.

Recent work by Youngstedt *et al* [42] investigated the effect of light therapy on combat related PTSD in a population of 69 Veterans, using 10,000 lux for 30 min/day for 4 weeks. This study demonstrated no change in anxiety, depression or sleep (via subjective means or objective actigraphy), but did show improvements in PTSD symptom severity that were correlated with phase advancements in via actigraphy derived circadian rhythm assessments. Although the present study also demonstrated an improvement in PTSD symptom severity, these studies diverge in sleep specific outcomes. Methodologically, the Youngstedt et al study was placebo controlled but employed a treatment duration of 30 minutes (rather than 60 minutes in the present study) and used the Pittsburgh Sleep Quality Index (rather than the ISI for subjective sleep changes). The combination of the shorter treatment duration and different validated questionnaire, may in part, explain differences in sleep specific outcomes. It is also possible that there were differences between the study by Youngstedt et al and the present study in both the presence of comorbid TBI and the spectral content of the light source employed, both of which were not reported.

### Limitations and further considerations

This study evaluated a single dose (10,000 lux), duration (60 minutes daily for 4-weeks), and timing (morning) of light therapy. There have been a plethora of studies using many differing parameters of light therapy, and these are generally common parameter ranges [64]. However, many studies do not follow the recommended guidelines for reporting of light exposure [51], making it difficult to know the amount and spectral content of the light reaching the eye, and nearly impossible to accurately compare findings across studies. A strength of our study is the rigorous assessment of light exposure from our protocol (S1 Table; **Supporting information**). Additionally, accurately recording adherence to the light therapy is an inherent challenge to this intervention [79] and a strength to this study is collecting both self-reported adherence and an objective surrogate via the actiwatch luxometer. Taken together, adherence for ~90% of the population was very high (60 minutes; 5–7 days per week). Nevertheless, subjects with less-than-ideal adherence did not differ from those with “perfect” adherence, and incorporating adherence as a statistical covariate did not change any of the reported outcomes.

This pilot study sought to determine the feasibility of conducting a larger clinical trial and as such, was not powered to detect treatment efficacy or changes in blood-based biomarker levels. While results support the feasibility, acceptability, and preliminary efficacy, including plasma biomarkers, conclusions cannot be made with regard to definitive mechanisms or directionality (e.g., sleep and mood, sleep and pain). Our data suggest advancing circadian phase to be a contributing factor, but secondary changes due to lights pleiotropic effects also likely contributed and cannot be ruled out. Instead of using mid-sleep time as a surrogate for circadian phase, serial serum or salivary melatonin collection to determine DLMO would be a more definitive biomarker with which to evaluate potential circadian effects. Actigraphy was collected and analyzed in 2 minute, rather than 1 minute, epochs (both of which are commonly reported in the literature [80]). An epoch duration of 2 minutes was chosen for the sake of preserving internal battery life and based on internal validation studies showing very little change in temporal resolution. Furthermore, because this study is single-arm and open-label, potential expectancy and/or placebo effects could not be determined, and the possibility remains that a change in daily routine, rather than the light therapy itself, improved sleep patterns. However, one piece of evidence arguing against this possibility is the lack of change in the Sleep Hygiene Index. Of note, approximately 50% of the subjects in this population reported use of sleep medications (no subjects reported melatonin usage). However, subjects did not change their sleep medication usage over the study period and no changes in statistical outcomes were found after controlling for medication usage.

Overall, this study provides valuable process validation and contributes towards the design of an ongoing randomized, single-blinded, placebo-controlled clinical trial examining sleep, mood, pain, and biomarker outcome measures (NCT03968874, sponsored by the Department of Defense and the Center for Neuroscience and Regenerative Medicine; and NCT03785600, sponsored by the Department of Veteran Affairs, Rehabilitation Research and Development Service).

## Conclusion

Morning bright light therapy was associated with improved sleep in Veterans with a history of mild TBI and showed high feasibility and moderate acceptability, as well as preliminary efficacy to improve other TBI sequela. Specific outcome measures improving post-light therapy included: subjective and objective sleep, symptoms related to TBI, PTSD, mood, and pain, and blood-based pro-inflammatory cytokines. Furthermore, comorbid PTSD significantly predicted improved insomnia post-light therapy. Overall, these results strongly support the rationale for a larger, blinded and placebo-controlled randomized clinical trial to more rigorously evaluate the efficacy of morning bright light therapy to improve sleep in Veterans with TBI.

## Supporting information

S1 Checklist CONSORT checklist. (DOC)

S1 Table Spectral power distribution data. (DOCX)

S1 File Lim_4085_Protocol. (DOCX)
