# Supplementary material for: Feasibility and preliminary efficacy for morning bright light therapy to improve sleep and plasma biomarkers in US Veterans with TBI. A prospective, open-label, single-arm trial
Source: PLoS One. 2022 Apr 14;17(4):e0262955. doi: 10.1371/journal.pone.0262955 (PMC9009710; doi:10.1371/journal.pone.0262955)
Supplement: S1 Table — (DOCX) [file pone.0262955.s002.docx]

| **Table S1**. *Spectral power distribution data*. | |
| --- | --- |
| Wave length (nm) | Spectral Irradiance (W/m^2^/nm) |
| 378 | 8.566E-05 |
| 379 | 7.864E-05 |
| 380 | 7.293E-05 |
| 381 | 7.927E-05 |
| 382 | 1.055E-04 |
| 383 | 1.334E-04 |
| 384 | 1.585E-04 |
| 385 | 1.689E-04 |
| 386 | 1.534E-04 |
| 387 | 1.343E-04 |
| 388 | 1.164E-04 |
| 389 | 1.103E-04 |
| 390 | 1.280E-04 |
| 391 | 1.526E-04 |
| 392 | 1.817E-04 |
| 393 | 2.107E-04 |
| 394 | 2.335E-04 |
| 395 | 2.574E-04 |
| 396 | 2.833E-04 |
| 397 | 3.102E-04 |
| 398 | 3.347E-04 |
| 399 | 3.674E-04 |
| 400 | 4.123E-04 |
| 401 | 4.759E-04 |
| 402 | 5.708E-04 |
| 403 | 6.835E-04 |
| 404 | 8.127E-04 |
| 405 | 9.542E-04 |
| 406 | 1.095E-03 |
| 407 | 1.254E-03 |
| 408 | 1.439E-03 |
| 409 | 1.654E-03 |
| 410 | 1.901E-03 |
| 411 | 2.191E-03 |
| 412 | 2.530E-03 |
| 413 | 2.924E-03 |
| 414 | 3.378E-03 |
| 415 | 3.902E-03 |
| 416 | 4.504E-03 |
| 417 | 5.192E-03 |
| 418 | 5.950E-03 |
| 419 | 6.819E-03 |
| 420 | 7.817E-03 |
| 421 | 8.961E-03 |
| 422 | 1.025E-02 |
| 423 | 1.172E-02 |
| 424 | 1.341E-02 |
| 425 | 1.535E-02 |
| 426 | 1.752E-02 |
| 427 | 2.000E-02 |
| 428 | 2.284E-02 |
| 429 | 2.607E-02 |
| 430 | 2.971E-02 |
| 431 | 3.383E-02 |
| 432 | 3.848E-02 |
| 433 | 4.372E-02 |
| 434 | 4.952E-02 |
| 435 | 5.602E-02 |
| 436 | 6.329E-02 |
| 437 | 7.142E-02 |
| 438 | 8.056E-02 |
| 439 | 9.070E-02 |
| 440 | 1.019E-01 |
| 441 | 1.141E-01 |
| 442 | 1.280E-01 |
| 443 | 1.430E-01 |
| 444 | 1.587E-01 |
| 445 | 1.749E-01 |
| 446 | 1.919E-01 |
| 447 | 2.088E-01 |
| 448 | 2.250E-01 |
| 449 | 2.398E-01 |
| 450 | 2.522E-01 |
| 451 | 2.621E-01 |
| 452 | 2.696E-01 |
| 453 | 2.744E-01 |
| 454 | 2.752E-01 |
| 455 | 2.726E-01 |
| 456 | 2.675E-01 |
| 457 | 2.603E-01 |
| 458 | 2.510E-01 |
| 459 | 2.402E-01 |
| 460 | 2.284E-01 |
| 461 | 2.161E-01 |
| 462 | 2.043E-01 |
| 463 | 1.931E-01 |
| 464 | 1.823E-01 |
| 465 | 1.719E-01 |
| 466 | 1.625E-01 |
| 467 | 1.539E-01 |
| 468 | 1.459E-01 |
| 469 | 1.384E-01 |
| 470 | 1.312E-01 |
| 471 | 1.245E-01 |
| 472 | 1.181E-01 |
| 473 | 1.122E-01 |
| 474 | 1.066E-01 |
| 475 | 1.014E-01 |
| 476 | 9.667E-02 |
| 477 | 9.239E-02 |
| 478 | 8.866E-02 |
| 479 | 8.551E-02 |
| 480 | 8.285E-02 |
| 481 | 8.067E-02 |
| 482 | 7.896E-02 |
| 483 | 7.772E-02 |
| 484 | 7.687E-02 |
| 485 | 7.638E-02 |
| 486 | 7.621E-02 |
| 487 | 7.631E-02 |
| 488 | 7.667E-02 |
| 489 | 7.725E-02 |
| 490 | 7.803E-02 |
| 491 | 7.897E-02 |
| 492 | 8.006E-02 |
| 493 | 8.129E-02 |
| 494 | 8.265E-02 |
| 495 | 8.412E-02 |
| 496 | 8.568E-02 |
| 497 | 8.731E-02 |
| 498 | 8.902E-02 |
| 499 | 9.077E-02 |
| 500 | 9.256E-02 |
| 501 | 9.437E-02 |
| 502 | 9.618E-02 |
| 503 | 9.796E-02 |
| 504 | 9.972E-02 |
| 505 | 1.014E-01 |
| 506 | 1.031E-01 |
| 507 | 1.047E-01 |
| 508 | 1.063E-01 |
| 509 | 1.077E-01 |
| 510 | 1.092E-01 |
| 511 | 1.105E-01 |
| 512 | 1.118E-01 |
| 513 | 1.130E-01 |
| 514 | 1.142E-01 |
| 515 | 1.153E-01 |
| 516 | 1.163E-01 |
| 517 | 1.172E-01 |
| 518 | 1.181E-01 |
| 519 | 1.190E-01 |
| 520 | 1.198E-01 |
| 521 | 1.206E-01 |
| 522 | 1.213E-01 |
| 523 | 1.220E-01 |
| 524 | 1.227E-01 |
| 525 | 1.233E-01 |
| 526 | 1.239E-01 |
| 527 | 1.244E-01 |
| 528 | 1.249E-01 |
| 529 | 1.254E-01 |
| 530 | 1.259E-01 |
| 531 | 1.263E-01 |
| 532 | 1.268E-01 |
| 533 | 1.272E-01 |
| 534 | 1.275E-01 |
| 535 | 1.279E-01 |
| 536 | 1.282E-01 |
| 537 | 1.286E-01 |
| 538 | 1.289E-01 |
| 539 | 1.292E-01 |
| 540 | 1.294E-01 |
| 541 | 1.297E-01 |
| 542 | 1.299E-01 |
| 543 | 1.302E-01 |
| 544 | 1.304E-01 |
| 545 | 1.306E-01 |
| 546 | 1.309E-01 |
| 547 | 1.311E-01 |
| 548 | 1.314E-01 |
| 549 | 1.316E-01 |
| 550 | 1.319E-01 |
| 551 | 1.321E-01 |
| 552 | 1.323E-01 |
| 553 | 1.325E-01 |
| 554 | 1.327E-01 |
| 555 | 1.328E-01 |
| 556 | 1.330E-01 |
| 557 | 1.331E-01 |
| 558 | 1.332E-01 |
| 559 | 1.333E-01 |
| 560 | 1.334E-01 |
| 561 | 1.334E-01 |
| 562 | 1.335E-01 |
| 563 | 1.336E-01 |
| 564 | 1.336E-01 |
| 565 | 1.336E-01 |
| 566 | 1.336E-01 |
| 567 | 1.336E-01 |
| 568 | 1.335E-01 |
| 569 | 1.335E-01 |
| 570 | 1.334E-01 |
| 571 | 1.333E-01 |
| 572 | 1.332E-01 |
| 573 | 1.330E-01 |
| 574 | 1.329E-01 |
| 575 | 1.327E-01 |
| 576 | 1.325E-01 |
| 577 | 1.322E-01 |
| 578 | 1.320E-01 |
| 579 | 1.317E-01 |
| 580 | 1.314E-01 |
| 581 | 1.310E-01 |
| 582 | 1.307E-01 |
| 583 | 1.303E-01 |
| 584 | 1.299E-01 |
| 585 | 1.295E-01 |
| 586 | 1.291E-01 |
| 587 | 1.286E-01 |
| 588 | 1.281E-01 |
| 589 | 1.276E-01 |
| 590 | 1.271E-01 |
| 591 | 1.265E-01 |
| 592 | 1.259E-01 |
| 593 | 1.253E-01 |
| 594 | 1.246E-01 |
| 595 | 1.239E-01 |
| 596 | 1.232E-01 |
| 597 | 1.225E-01 |
| 598 | 1.217E-01 |
| 599 | 1.209E-01 |
| 600 | 1.201E-01 |
| 601 | 1.193E-01 |
| 602 | 1.184E-01 |
| 603 | 1.175E-01 |
| 604 | 1.166E-01 |
| 605 | 1.157E-01 |
| 606 | 1.147E-01 |
| 607 | 1.137E-01 |
| 608 | 1.126E-01 |
| 609 | 1.115E-01 |
| 610 | 1.104E-01 |
| 611 | 1.093E-01 |
| 612 | 1.081E-01 |
| 613 | 1.070E-01 |
| 614 | 1.058E-01 |
| 615 | 1.046E-01 |
| 616 | 1.034E-01 |
| 617 | 1.021E-01 |
| 618 | 1.009E-01 |
| 619 | 9.958E-02 |
| 620 | 9.828E-02 |
| 621 | 9.695E-02 |
| 622 | 9.562E-02 |
| 623 | 9.427E-02 |
| 624 | 9.291E-02 |
| 625 | 9.154E-02 |
| 626 | 9.016E-02 |
| 627 | 8.877E-02 |
| 628 | 8.735E-02 |
| 629 | 8.592E-02 |
| 630 | 8.449E-02 |
| 631 | 8.306E-02 |
| 632 | 8.163E-02 |
| 633 | 8.019E-02 |
| 634 | 7.876E-02 |
| 635 | 7.733E-02 |
| 636 | 7.589E-02 |
| 637 | 7.446E-02 |
| 638 | 7.304E-02 |
| 639 | 7.163E-02 |
| 640 | 7.024E-02 |
| 641 | 6.886E-02 |
| 642 | 6.748E-02 |
| 643 | 6.611E-02 |
| 644 | 6.474E-02 |
| 645 | 6.339E-02 |
| 646 | 6.204E-02 |
| 647 | 6.071E-02 |
| 648 | 5.940E-02 |
| 649 | 5.810E-02 |
| 650 | 5.682E-02 |
| 651 | 5.556E-02 |
| 652 | 5.431E-02 |
| 653 | 5.308E-02 |
| 654 | 5.186E-02 |
| 655 | 5.065E-02 |
| 656 | 4.945E-02 |
| 657 | 4.827E-02 |
| 658 | 4.711E-02 |
| 659 | 4.597E-02 |
| 660 | 4.485E-02 |
| 661 | 4.375E-02 |
| 662 | 4.268E-02 |
| 663 | 4.162E-02 |
| 664 | 4.057E-02 |
| 665 | 3.955E-02 |
| 666 | 3.854E-02 |
| 667 | 3.756E-02 |
| 668 | 3.661E-02 |
| 669 | 3.567E-02 |
| 670 | 3.475E-02 |
| 671 | 3.385E-02 |
| 672 | 3.296E-02 |
| 673 | 3.209E-02 |
| 674 | 3.124E-02 |
| 675 | 3.040E-02 |
| 676 | 2.957E-02 |
| 677 | 2.876E-02 |
| 678 | 2.797E-02 |
| 679 | 2.720E-02 |
| 680 | 2.644E-02 |
| 681 | 2.571E-02 |
| 682 | 2.500E-02 |
| 683 | 2.430E-02 |
| 684 | 2.363E-02 |
| 685 | 2.297E-02 |
| 686 | 2.233E-02 |
| 687 | 2.169E-02 |
| 688 | 2.107E-02 |
| 689 | 2.046E-02 |
| 690 | 1.987E-02 |
| 691 | 1.930E-02 |
| 692 | 1.876E-02 |
| 693 | 1.823E-02 |
| 694 | 1.771E-02 |
| 695 | 1.721E-02 |
| 696 | 1.671E-02 |
| 697 | 1.623E-02 |
| 698 | 1.576E-02 |
| 699 | 1.529E-02 |
| 700 | 1.483E-02 |
| 701 | 1.438E-02 |
| 702 | 1.395E-02 |
| 703 | 1.353E-02 |
| 704 | 1.313E-02 |
| 705 | 1.273E-02 |
| 706 | 1.235E-02 |
| 707 | 1.196E-02 |
| 708 | 1.157E-02 |
| 709 | 1.119E-02 |
| 710 | 1.082E-02 |
| 711 | 1.048E-02 |
| 712 | 1.016E-02 |
| 713 | 9.847E-03 |
| 714 | 9.549E-03 |
| 715 | 9.253E-03 |
| 716 | 8.962E-03 |
| 717 | 8.680E-03 |
| 718 | 8.406E-03 |
| 719 | 8.147E-03 |
| 720 | 7.898E-03 |
| 721 | 7.656E-03 |
| 722 | 7.417E-03 |
| 723 | 7.171E-03 |
| 724 | 6.926E-03 |
| 725 | 6.687E-03 |
| 726 | 6.458E-03 |
| 727 | 6.251E-03 |
| 728 | 6.058E-03 |
| 729 | 5.873E-03 |
| 730 | 5.691E-03 |
| 731 | 5.501E-03 |
| 732 | 5.312E-03 |
| 733 | 5.128E-03 |
| 734 | 4.950E-03 |
| 735 | 4.787E-03 |
| 736 | 4.632E-03 |
| 737 | 4.485E-03 |
| 738 | 4.343E-03 |
| 739 | 4.203E-03 |
| 740 | 4.068E-03 |
| 741 | 3.939E-03 |
| 742 | 3.819E-03 |
| 743 | 3.717E-03 |
| 744 | 3.621E-03 |
| 745 | 3.527E-03 |
| 746 | 3.431E-03 |
| 747 | 3.315E-03 |
| 748 | 3.199E-03 |
| 749 | 3.087E-03 |
| 750 | 2.982E-03 |
| 751 | 2.903E-03 |
| 752 | 2.831E-03 |
| 753 | 2.764E-03 |
| 754 | 2.697E-03 |
| 755 | 2.621E-03 |
| 756 | 2.547E-03 |
| 757 | 2.474E-03 |
| 758 | 2.406E-03 |
| 759 | 2.346E-03 |
| 760 | 2.289E-03 |
| 761 | 2.231E-03 |
| 762 | 2.169E-03 |
| 763 | 2.095E-03 |
| 764 | 2.023E-03 |
| 765 | 1.956E-03 |
| 766 | 1.905E-03 |
| 767 | 1.876E-03 |
| 768 | 1.853E-03 |
| 769 | 1.829E-03 |
| 770 | 1.791E-03 |
| 771 | 1.733E-03 |
| 772 | 1.675E-03 |
| 773 | 1.622E-03 |
| 774 | 1.591E-03 |
| 775 | 1.585E-03 |
| 776 | 1.586E-03 |
| 777 | 1.587E-03 |
| 778 | 1.576E-03 |
| 779 | 1.553E-03 |
| 780 | 1.523E-03 |
