# Supplementary material for: Feasibility and preliminary efficacy for morning bright light therapy to improve sleep and plasma biomarkers in US Veterans with TBI. A prospective, open-label, single-arm trial
Source: PLoS One. 2022 Apr 14;17(4):e0262955. doi: 10.1371/journal.pone.0262955 (PMC9009710; doi:10.1371/journal.pone.0262955)
Supplement: S1 File — (DOCX) [file pone.0262955.s003.docx]

**Research Protocol/Protocol Addendum Template**

**Title**

Morning bright light to improve sleep quality in Veterans

**Investigators**

Principal Investigator: Miranda Lim, M.D., Ph.D.

**Specific Aims/Purpose**

This proposal aims to examine the relationship between morning bright light (MBL) exposure and sleep disturbances in Veterans.

**Specific Aim 1:** Determine the relationship between MBL exposure on overall daily function and quality of life in Veterans.

**Specific Aim 2:** Determine the relationship between MBL on objective sleep quality in Veterans.

**Scientific Rationale and Significance**

Sleep disturbances are highly prevalent among Veterans, and often negatively impact daily functioning and quality of life. There are many reasons why sleep disturbances are so common among Veterans, including prior exposure to physical and psychological trauma in combat (e.g. traumatic brain injury – TBI, post-traumatic stress disorder – PTSD), a high rate of mood disorders and increased risk for neurodegeneration, among neuropsychiatric illnesses in which sleep disturbances are a common or core feature.^1-8^

Chronic sleep disturbances have been associated with impaired cognition, negative mood, impaired emotional processing, heightened pain sensitivity, and decreased quality of life^9-11^. Poor sleep quality and sleep disturbances exacerbate symptoms of TBI and/or PTSD,^2^ impact the individual’s ability to cope with these symptoms,^3^ worsen neuropsychiatric symptoms of TBI and/or PTSD,^4^ and inhibit full participation in rehabilitation programs.^5^ Furthermore, sleep plays an important role in memory consolidation, synaptic plasticity and neurogenesis.^6^ Adequate sleep is critical for optimal functioning in healthy adults.^7^ A relationship between poor sleep quality and depression suggests that sleep disturbances may be both a consequence and an exacerbating factor of depression.^8^

Light exposure from either the sun or alternative methods that mimic natural light, particularly in the morning hours, has been shown to improve sleep quality in a variety of populations. Ocular light exposure can be both a stimulant and an antidepressant, eliciting circadian, neuroendocrine, and neurobehavioral responses. These “non-visual” responses include synchronization of the circadian clock^9^ via regulating melatonin secretion,^10^ acute alerting effects,^11,12^ and mood enhancement.^13^ Indeed, daily bright light exposure has been shown to improve depressive symptoms in seasonal affective disorder.^14^ Subjective improvements in fatigue have also been noted in patients with cancer with increasing exposure to light,^15^ as well as in healthy office workers exposed to bright light.^16,17^

In a previous pilot study in civilians with and without TBI (*n* = 10) who reported sleep disturbances, morning bright light (MBL; <1000 lux) for 45 min/day for 4-weeks decreased fatigue and daytime sleepiness, and showed a trend toward improved self-reported depression and psychomotor vigilance performance. MBL has also improved sleep disturbances, cognition, and behavioral disturbances in elderly patients (~60-90 years of age) with a variety of neurodegenerative conditions including dementia, Alzheimer’s, Huntington’s, and Parkinson’s disease.^18–20^ Thus, there is substantial scientific evidence that there is a strong relationship between MBL exposure and sleep quality/quality of life. However, to date, no large scale studies have examined this relationship in a Veteran population, which is enriched for sleep disturbances and many neuropsychiatric comorbidities.

Therefore, we propose to examine the relationship between light exposure and sleep quality and functional outcomes in a large cohort of Veterans, many of whom have been diagnosed with TBI and/or PTSD. Results from this study could potentially inform a larger future controlled treatment trial utilizing light therapy for the treatment of sleep disturbances in TBI and/or PTSD.

**Research Design and Methods**

Veterans within the VA Portland Healthcare System (VAPORHCS) who have been referred to the VA Sleep Disorders Clinic or related outpatient clinics will be recruited for participation in this exploratory, prospective, single-arm open-label trial. For details regarding the informed consent process, inclusion/exclusion criteria, and specific recruitment strategies, see *Study Population* and *Subject Identification/Recruitment* sections below.

After providing written and verbal informed consent, all participants will be asked to wear an actigraphy watch (Actiwatch 2 by Philips Respironics) with a luxometer for at ~35 days in order to measure light exposure during the day and night. Actigraphy watches also contain an accelerometer to measure activity, and have been validated against the gold standard in-lab polysomnography in several populations (including those with chronic insomnia and depression).^21^ Watches are user friendly, requiring no user input to successfully operate. Study personnel will provide the actigraphy watch fully charged and configured.

After 7 days of baseline whereby participants do not engage in MBL exposure, participants will begin the intervention period. This involves using a provided lightbox (LightPad mini, Aurora Light Solutions) in the morning upon waking for 60 minutes each morning for 28 days.

This lightbox is the same brand lightbox that is prescribed by VAPORHCS sleep clinicians for patients with seasonal affective disorder or circadian rhythm sleep disorders. This lightbox is very user friendly, with a single on/off button, and is lightweight and portable (9 in. wide and 5 in. tall).

Participants will be educated in principles of optimal sleeping habits (i.e., sleep hygiene). Poor sleep hygiene is well established to negatively impact sleep quality (e.g., drinking caffeine before bedtime), and improving sleep hygiene has been shown to improve sleep quality in patients with sleep disturbances secondary to poor sleep hygiene.^22^

Clinical intake questionnaires (administered to all patients in the Sleep Clinic in the waiting room) will be collated for consented participants and analyzed as part of this research protocol. The clinical intake questionnaire takes ~35 minutes to fill out, and includes self-reported measures on sleep history, sleep quality, sleep hygiene, past medical history, social history, quality of life, mental health outcomes, and other metrics that are used clinically to evaluate and treat patients with sleep complaints.

Pre-intervention will include standard phlebotomy, performed at the VA Phlebotomy Lab. Phlebotomists will draw 2-3 Lavender top (appropriate for plasma separation) 7 ml tubes, and 2-3 Tiger top (appropriate for serum separation) 7 ml tubes. In total, ~45 ml of whole blood will be removed (~3 tablespoons). The lab will notify study personnel once ready for pickup (1-2 minute walk from our laboratory). Study personnel will invert blood samples 10x to ensure thorough mixing, spin down whole blood cells, and separate plasma/serum. Aliquots will then be stored at -80^o^C in a locked freezer inside of a locked laboratory and VA PIV badge secured research area (Building 101, 5^th^ floor). Samples will be coded and labeled with only a participant ID when stored. Data obtained from these samples will be used exclusively for research purposes. Upon receipt of the blood samples, participants will receive a $10 Fred Meyer gift card.

There will be follow-up calls to all participants approximately weekly after consent and start of the study. These calls will be made to inquire about participants’ experiences with light exposure, use of equipment, to provide assistance with given equipment, and to answer any general questions about the study that they have.

At the end of participants study period (i.e., 28 days of 60 minutes of MBL exposure), participants will be asked to complete a post-study questionnaire. This post-study questionnaire will include self-reported measures on sleep history, sleep quality, sleep hygiene, past medical history, social history, quality of life, mental health outcomes, and other metrics that affect sleep. Participants will be asked to return the equipment to study personnel at this time point.

The follow-up questionnaire will be mailed to each participant alongside a prepaid return mailer for both the questionnaires and any remaining equipment. Participants will also be given the option to return the equipment to study personnel on-site. In this instance, the follow-up questionnaire will be completed in-person at the time of equipment return. Participants will receive $70 worth in Fred Meyer gift cards when their equipment and completed follow-up questionnaires have been returned. Finally, participants will be invited to provide a post-study blood sample in the same manner and conditions as described earlier. Participants providing a post-study blood sample will also receive a $10 Fred Meyer gift card.

For any questionnaires that are not returned throughout the duration of the study, approved study personnel may call participants to ask about the status of the questionnaires. If a participant needs a questionnaire mailed again due to an event such as mail failure or loss of the questionnaire, or if a participant prefers to answer questions in person or by phone, respective steps to accommodate these requests will be considered.

Participants who agree to participate in this study will be asked their permission to include the study data, blood samples, and contact information collected in this study in a long-term data repository from which other research questions about sleep disorders and functional outcomes might be studied. The data repository will be housed at VAPORHCS and accessed through VA computers secured by encryption and password protection.

**Study Population**

**A. Number of Subjects.**

This single-arm open-label trial is seeking to recruit 25-50 participants with TBI. Thus, it is anticipated we may screen 40-75 subjects in order to meet all eligibility criteria (see below). This sample size was calculated from a power analysis (see *Power Analysis* below) based on previously published studies. In the event there is difficulty recruiting for this study, the participants in Dr. Miranda Lim’s current IRB-approved data repository (IRB #3636) have given permission to be re-contacted for future studies and may initially be mailed a letter to gauge interest.

**B. Inclusion and Exclusion Criteria.**

Inclusion:

- Veterans (male and female)
- TBI documented via the Head Trauma Events Checklist

Exclusion:

- Non-Veteran
- Non-English speaking
- Decisionally-impaired
- Macular degeneration
- Bipolar disorder
- Currently using a lightbox

Our exclusion criteria will not exclude any specific class of persons who might benefit from the proposed research. Every effort will be made to include women and minority groups in this study.

**C. Power Analysis.**

The proposed number of Veterans is based on *a priori* power analyses from previous work investigating the effect of MBL to mitigate sleep problems^23^ with α set to <0.05 and β set to <0.20 (i.e., power >0.80). Thus, the probability of making a type I or type II error will be controlled to be <5% and <20%, respectively.

**Subject Identification/Recruitment**

Veterans (male and female) enrolled in the VAPORHCS will be recruited by the Study Coordinator from the VAPORHCS Sleep Disorders Clinic, or other outpatient clinics at VAPORHCS that have referred participants to us Veterans will be approached in person, evaluated for eligibility in this study, and if interested, invited to undergo verbal and written informed consent.

Flyers will also be used to recruit Veterans at VAPORHCS. These flyers will not be posted, but instead will be given to clinicians at outpatient clinics to give to interested subjects. Interested subjects who contact us will then be screened over the phone with our phone script. If the Veteran is eligible and interested after the phone screen, we will plan to meet in person at VAPORHCS to get written and verbal informed consent.

Only Veterans who are physically and mentally able to provide written and verbal informed consent and travel to and from the VAPORHCS will be included in this study.

We will draw on established recruiting methods, which we have successfully used for the past two years at VAPORHCS. These strategies ensure planned representation across sex, race, and ethnic groups. A previous study of similar size is currently underway in which approximately 670 Veterans have already been consented and will be enrolled in the VAPORHCS Sleep Disorders Data Repository (IRB #3636, #3641; PI Miranda Lim) over the course of two years of recruitment, demonstrating feasibility of this approach. The participants in said repository have agreed to be recontacted for their interest in future studies. In the event that difficulty arises recruiting participants for the proposed study, participants from the repository will be contacted and inquired about their interest in participating for this study as well.

**Informed Consent**

All informed consent processes will be completed by trained study staff. Participants will be recruited from the VAPORHCS Sleep Disorders Clinic or from other related outpatient clinics. In the VAPORHCS Sleep Disorders Clinic, participants will be referred by a clinician upon arrival for their clinically indicated overnight sleep testing. This clinic appointment consists of 30-90 minutes of a clinical intake, watching an educational video, and waiting for a sleep technician to apply electrodes for their overnight study. Patients are told by clinical staff to arrive 30 minutes earlier than their scheduled appointment times and to wait in the waiting room during this time. During this waiting period, potential participants will be approached to gauge their interest in the study by trained study staff. The study goals, procedures, and informed consent will be communicated verbally in person, while referring to the written informed consent document. Participants will be given an opportunity to have all questions answered before they provide verbal and written consent. Additionally, if participants determine that they do not have adequate time at this first visit, they are welcome to return to complete the consent process and pick up equipment and usage instructions at a later date. Written informed consent will be obtained from all participants prior to their participation, provided they meet all inclusion/exclusion criteria. Each participant will be told that he/she will be free to discontinue participation in the experiment at any time and that the investigators reserve the right to discontinue the research protocol at any time.

**Risks and Side Effects:**

The delivery of usual care in the Sleep Disorders Clinic is not altered in this study and therefore poses no additional risk in the clinical overnight sleep test. This study otherwise carries minimal risk as it is primarily observational through the use of collected self-report measures and actigraphy/luxometer data.

**Psychological Risks:** Differing from usual care are the follow-up symptom assessments and permission to store data, blood samples, and contact information from this study in a data repository. The questionnaires take approximately 20 minutes to complete. Some of the questions might cause psychological distress due to an inquiry of social and medical history. Participants will be reminded that they can choose to not answer any of the questions and opt out of the study at any time. Since questions are asked about mood, including thoughts of self-harm and depression, there is a possibility that information about thoughts of suicide or evidence of depression will be gained that will require an intervention. How this information will be handled is discussed below in the *Suicidality* section.

**Physical Risks:** Light from a lightbox is essentially indistinguishable from natural light from the sun and consists of broad spectrum white light. Morning bright light from a lightbox poses very minimal and rare risks. One study showed that light intensity of 10,000 lux received for an average of 40+ minutes per day over a five-year period had no major side effects.^24^ Eye irritation, irritability, headache, nausea, sensation of glare, dryness of eyes, and dryness of skin are some of the potential minor risks from lightbox use (as can be from natural light exposure from the sun), and can be avoided by limiting the amount of time exposed to light. A rare occurrence of mania or rapid-cycling may occur in those with Bipolar Disorder due to too much exposure to bright light. For this reason, this study will exclude those with Bipolar Disorder. The manufacturer notes in the user guide that people with macular degeneration may be more at risk of retinal damage from blue light and should avoid it. Those with macular degeneration will be excluded from the study, as stated above in *Inclusion and Exclusion Criteria*. If problems are experienced, participants are free to stop the study at any time and/or call the Study Coordinator with any questions or issues. Participants may experience skin irritation from the actigraphy watch wristband, although very uncommon. Participants will be told to remove the actigraphy watch if this occurs. Participants may also feel some pain from the needle when their blood is drawn. There is a small chance that the needle will cause bleeding, a bruise, or an infection at the draw site. Participants will be reminded that they can choose not to have their blood drawn and opt out of the study at any time.

**Other Risks:** Because this study collects protected health information, there is always a risk of a breach of confidentiality. This risk is addressed below under *Privacy and Confidentiality*.

The possible benefits to the participants and to future understanding and improvements in delivery of sleep-related care to Veterans are reasonable and outweigh the risks of minor and rare physical harm, emotional distress, or breach of confidentiality to study participants.

**Participant Safeguards:**

This study will not include any vulnerable populations. Potential participants will be gauged for their interest in the study prior to being handed a consent form. Those participants that can verbally acknowledge understanding of the study and agree to participate will be consented by the Study Coordinator. If the Study Coordinator detects decisional impairment or inability to understand the study, such as repeating back the basic concepts of the study, that person will not be included to participate.

**Suicidality*:***

In the event that a Veteran expresses the thought of harming themselves when they are at the clinic for the overnight sleep test, the sleep lab technician or the researcher, if present, will accompany the Veteran to the Emergency Department for a warm-transfer.

If during a follow-up phone call after the sleep test the Veteran expresses thoughts of harming themselves, then the researcher will contact the VA national crisis line at 585-393-7938 or 1-800-273-8255 or the local suicide prevention line at x52857 and connect the Veteran to that help by phone. In the event a direct phone transfer cannot be completed, then the researcher will ask that the participant remain available by phone. The researcher will provide the VA National Suicide Prevention Hotline with the name and phone of the participant and the VA Hotline will then phone the participant. The researcher will follow-up afterwards with the participant by phone to ensure that the participant has received adequate help and is safe.

In the event that questionnaire answers indicate depression or suicidality, their primary mental health provider will be notified. If they do not have a mental health provider, then their primary care physician will be notified to make a referral.

**Benefits:**

Participants may directly benefit from this study through the designated morning bright light and/or sleep hygiene education that is offered. Affected outcomes might include improved quality of sleep, an increase in quality of life, improved cognitive functioning, and reduction of pain.

See *Subject Compensation* section below for modest compensation given to participants.

**Protected Health Information:**

The following protected health information (PHI) will be collected in this study: history regarding diagnosis of a sleep disorder; medical history will be confirmed or examined in chart if TBI, PTSD, and/or depression is indicated on surveys. In addition, information collected from symptom questionnaires at clinical intake will include PHI such as name, mailing address(es), email addresses, and phone number on the cover page. Follow-up survey questionnaires on symptoms related to sleep, mood, trauma, and social history will be collected on paper copies with a participant ID number and no identifiable data. The original questionnaires will be stored in a locked file cabinet, in a locked office, on the 4^th^ floor of VAPORHCS in building 101, room 432A. This data will also be entered into a HIPAA-secure REDCap database housed at OHSU. Original consent and authorization forms will be stored in a locked drawer and office (building 100, room 6C-145).

**Resources Available**

The VAPORHCS Sleep Disorders Clinic is the sponsor of this study. The Principal Investigator, Miranda Lim, M.D., Ph.D., is a clinical provider in the Sleep Disorders Clinic. There is office space available at the VAPORHCS Sleep Disorders Clinic (building 100-6C) and provided by Research Service (building 101, room 432A) with locked file drawers in a dedicated, locked room.

**Costs to Subjects:**

Participants will incur a minor cost from operating the energy-efficient lightboxes. The estimated electrical cost to power the lightbox amounts to <15 cents over the course of the study period. There are no other costs involved with participating in the study.

**Subject Compensation:**

Participants will receive a $10 Fred Meyer gift card upon receipt of each blood sample. Participants will receive $70 worth of Fred Meyer gift cards after returning equipment and completing the post-study questionnaire. Participants will also receive a $10 Fred Meyer gift card for the completion of each follow-up questionnaire.

**Privacy and Confidentiality:**

All hard copies of study documents will be stored in a locked file cabinet, in a locked room, identified with a study ID and not the participant’s name.

The study investigator providing informed consent may be a health care provider in the VAPORHCS Sleep Disorders Clinic, but patients who receive a sleep study with VAPORHCS do not need to participate in this study. The VAPORHCS Sleep Disorders Clinic has a high volume of patients, and so there is no need for coercion or undue influence to participate in this study.

To protect privacy and confidentiality, the electronic master list of study participants and their study identification number will be stored on the VAPORHCS limited-access Research drive and protected by password access to the VA computer system. Only IRB-approved study personnel will have access to this file. Electronic study data from the questionnaires will be stored in a HIPAA-secure REDCap database on the OHSU server.

**Information and/or Specimen Management**

Study information will be shared outside VAPORHCS to the affiliated university, OHSU. Information collected from symptom questionnaires at clinical intake will include PHI such as name, mailing address, and phone number on the cover page. Follow-up questionnaires will be collected on physical copies with no identifiable data and only a coded participant ID on the cover page. The original hard copy questionnaires, as well as original consent and authorization forms, will be stored in a locked file cabinet in a locked office on the 4^th^ floor of VAPORHCS in building 101, room 432A. These data will also be entered into a secure REDCap database housed on the OHSU server.

The actigraphy watches will be used to collect actigraphy data and will contain sleep patterns, overall light intake, and overall activity levels. The actigraphy watch has no ability to transmit wirelessly and must be connected to a charging dock to retrieve the data. Data will be downloaded onto a password-protected VA computer containing VA-approved proprietary software for actigraphy devices, accessible only behind a VA firewall. Actigraphy data will be summarized and added to the OHSU REDCap database. The raw actigraphy data will also be housed on the limited-access Research drive at VAPORHCS.

**Data and Safety Monitoring Plan**

It is very unlikely that study outcomes will adversely affect the health or well-being of research participants in this experimental study. Participants are under usual care for all treatment they receive for their sleep disorder in addition to the proposed study arms. If depression or risk of suicide are evident due to questionnaire data collected, then participants and possibly their health care provider will be informed about that information. This is discussed in the sections on *Suicidality* and on *Risks and Side Effects*.

**Step-by-Step Guidance on Conducting the Study**

1. Approach potential participants in the Sleep Disorders Clinic that are present for their clinically-indicated overnight sleep study and gauge interest. Consent the interested persons unless observed as decisionally-impaired or otherwise meeting exclusion criteria for entry.
2. Collate clinical intake questionnaires from the consented participants, offer a lightbox and actigraphy watch to each participant. Administer sleep hygiene education to all participants. Direct participants to phlebotomy lab for their pre-study blood draw.
3. Administer follow-up calls approximately weekly to each participant to check in and answer any questions participants may have regarding the study or equipment.
4. At least 30 days after consent, send post-study questionnaires to each participant with a prepaid return envelope. Alternatively, participants may decide to have the Study Coordinator call and answer questions by phone or complete the questionnaire on-site.
5. At 2-3 follow-up time points after enrollment, follow-up questionnaires will be sent to each participant with a prepaid return envelope. Alternatively, participants may decide to have the Study Coordinator call and answer questions by phone or complete the questionnaires on-site.
6. Participants who agree to long-term use of data collected in this study will have their study data, blood samples, and contact information from this study added to the VAPORHCS Sleep Disorders Data Repository (IRB#4086).

**References & Literature Cited**

1. Okie, S. Traumatic brain injury in the war zone. *N. Engl. J. Med.* **352,** 2043–2047 (2005).

2. Ouellet, M.-C. & Morin, C. M. Efficacy of Cognitive-Behavioral Therapy for Insomnia Associated With Traumatic Brain Injury. *Arch. Phys. Med. Rehabil.* **88,** 1581–1592 (2007).

3. Lew, H. L. *et al.* Prevalence of chronic pain, posttraumatic stress disorder, and persistent postconcussive symptoms in OIF/OEF veterans: polytrauma clinical triad. *J. Rehabil. Res. Dev.* **46,** 697–702 (2009).

4. Rao, V., McCann, U., Han, D., Bergey, A. & Smith, M. T. Does acute TBI-related sleep disturbance predict subsequent neuropsychiatric disturbances? *Brain Inj.* **28,** 20–6 (2014).

5. Worthington, A. D. & Melia, Y. Rehabilitation is compromised by arousal and sleep disorders: results of a survey of rehabilitation centres. *Brain Inj.* **20,** 327–332 (2006).

6. Abel, T., Havekes, R., Saletin, J. M. & Walker, M. P. Sleep, plasticity and memory from molecules to whole-brain networks. *Curr. Biol.* **23,** R774–R788 (2013).

7. Basner, M., Rao, H., Goel, N. & Dinges, D. F. Sleep Deprivation and Neurobehavioral Dynamics. *Curr. Opin. Neurobiol.* **23,** 854–863 (2013).

8. Fogelberg, D. J., Hoffman, J. M., Dikmen, S., Temkin, N. R. & Bell, K. R. Association of sleep and co-occurring psychological conditions at 1 yr after traumatic brain injury. *Arch. Phys. Med. Rehabil.* **93,** 1313–8 (2012).

9. Czeisler, C. A. *et al.* Bright Light Induction of Strong (Type 0) Resetting of the Human Circadian Pacemaker. *Science (80-. ).* **244,** 1328–1333 (1989).

10. Lewy, A. J., Wehr, T. A., Goodwin, F. K., Newsome, D. A. & Markey, S. P. Light Suppresses Melatonin Secretion in Humans. **210,** 1267–1269 (2010).

11. Lockley, S. W. & Gooley, J. J. Circadian Photoreception: Spotlight on the Brain. *Curr. Biol.* **16,** 795–797 (2006).

12. Cajochen, C. Alerting effects of light. *Sleep Med. Rev.* **11,** 453–464 (2007).

13. Golden, R. N. *et al.* The efficacy of light therapy in the treatment of mood disorders: A review and meta-analysis of the evidence. *Am. J. Psychiatry* **162,** 656–662 (2005).

14. Glickman, G., Byrne, B., Pineda, C., Hauck, W. W. & Brainard, G. C. Light therapy for Seasonal Affective Disorder with blue narrow-band light-emitting diodes (LEDs). *Biol. Psychiatry* **59,** 502–507 (2006).

15. Jeste, N. *et al.* Prevention of quality-of-life deterioration with light therapy is associated with changes in fatigue in women with breast cancer undergoing chemotherapy. *Qual. Life Res.* **22,** 1239–1244 (2013).

16. Viola, A. U., James, L. M., Schlangen, L. J. M. & Dijk, D. J. Blue-enriched white light in the workplace improves self-reported alertness, performance and sleep quality. *Scand. J. Work. Environ. Heal.* **34,** 297–306 (2008).

17. Mills, P. R., Tomkins, S. C. & Schlangen, L. J. M. The effect of high correlated colour temperature office lighting on employee wellbeing and work performance. *J. Circadian Rhythms* **5,** 2 (2007).

18. Peter-Derex, L., Yammine, P., Bastuji, H. & Croisile, B. Sleep and Alzheimer’s disease. *Sleep Med. Rev.* **19,** 29–38 (2015).

19. Iranzo, A. Sleep in Neurodegenerative Diseases. *Sleep Med. Clin.* **11,** 1–18 (2016).

20. Riemersma-van der Lek, R. F. *et al.* Effect of Bright Light and Melatonin on Cognitive and Noncognitive Function in Elderly Residents of Group Care Facilities. *JAMA* **299,** 2642–2655 (2008).

21. Marino, M. *et al.* Measuring sleep: accuracy, sensitivity, and specificity of wrist actigraphy compared to polysomnography. *Sleep* **36,** 1747–55 (2013).

22. Stepanski, E. J. & Wyatt, J. K. Use of sleep hygiene in the treatment of insomnia. *Sleep Med. Rev.* **7,** 215–225 (2003).

23. Alessi, C. A. *et al.* Randomized, controlled trial of a nonpharmacological intervention to improve abnormal sleep/wake patterns in nursing home residents. *J. Am. Geriatr. Soc.* **53,** 803–810 (2005).

24. Chesson, A. L. *et al.* Practice Parameters for the Use of Light Therapy in the Treatment of Sleep Disorders. *Sleep* **22,** 641–660 (1999).
